# A physics-informed neural network approach for estimating population-level pharmacokinetic parameters from aggregated concentration data

**DOI:** 10.1007/s10928-026-10019-w

**Published:** 2026-02-16

**Authors:** Periklis Tsiros, Vasileios Minadakis, Haralambos Sarimveis

**Affiliations:** School of Chemical Engineering, National Technical University of Athens, 9 Iroon Polytechniou Str, Zografou Campus, 15772 Athens Greece

**Keywords:** PINNS, Statistical modelling, ODEs, Pharmacokinetics

## Abstract

The pharmacokinetic literature is rich in aggregated concentration data that contain valuable information, yet tools to extract this information remain limited. This work introduces distributional physics-informed neural networks (D-PINNs), a novel algorithm designed to enable statistical modelling within the PINN framework, allowing recovery of pharmacokinetic parameter distributions at the population level from published concentration means and variances. Unlike traditional PINNs, which often focus on point estimates, D-PINNs incorporate distributional assumptions directly into the optimisation process. The framework utilises neural networks for predicting the mean and variance of the concentration over time. These predictions are then incorporated into a sampling-based procedure within the residual network, which uses the governing ordinary differential equation (ODE) system to compute the physics-informed loss term. The methodology accounts for both interindividual variability through the parameter distribution and measurement noise through a residual error model. The capability of D-PINNs to infer population-level parameter distributions from concentration summary statistics was demonstrated through a simple proof-of-concept using simulated data from a one-compartment pharmacokinetic model of intravenous drug administration. The model achieved high accuracy in estimating both the parameter distribution and the residual error. Hyperparameter tuning highlighted important aspects of model development. The modelling framework was then applied to real-world data to demonstrate its ability to recover information on the distribution of kinetic parameters in the studied population. Specifically, a minimal physiologically-based pharmacokinetic (mPBPK) model for monoclonal antibodies (mAbs) was fitted to aggregated plasma concentration data reported in the literature using D-PINNs. The same aggregated data were also analysed using a Markov chain Monte Carlo (MCMC) analogue to benchmark the proposed methodology.

## Introduction

Physics-informed neural networks (PINNs), introduced by Raissi et al. [1], have rapidly gained traction in a wide range of computational problems. The property that distinguishes PINNs from other deep learning algorithms is their dual network architecture, where a conventional neural network (NN) approximates the solution, while an auxiliary residual network encodes the governing differential equations of the physical system. This design allows PINNs to address both forward and inverse problems. In forward-problem settings, the differential equations and system parameters are known, and the network is used to predict the system’s state variables. In inverse problems, the parameters of the system are unknown and are inferred from observed state-variable data. The effectiveness of PINNs has been demonstrated across numerous applications, particularly in the field of computational fluid dynamics [1–8]. In the field of pharmacokinetics, the use of PINNs remains limited, with only a few studies showcasing their potential for estimating parameters and predicting system dynamics [9, 10].

Moving beyond purely deterministic approaches, accurately capturing and quantifying the variability and uncertainty in physical systems is often essential for making robust predictions and informed decisions. The parameters of a physical system have inherent variability that is propagated to the system’s response through the underlying system dynamics, which are expressed through mathematical equations. In fact, in several disciplines, understanding the variability of a parameter can be more significant than merely estimating its central tendency. In pharmacokinetics, the population-based analysis of interindividual variability is crucial [11]. The gold standard for estimating population variability in pharmacokinetics-pharmacodynamics systems is nonlinear mixed-effects (NLME) modeling, which quantifies how individuals deviate from the population mean and accounts for both biological and observational uncertainty [12]. Common estimation methods within the NLME framework include the First-Order Conditional Estimation (FOCE) method [13], the Stochastic Approximation Expectation-Maximization (SAEM) [14] and fully Bayesian Markov chain Monte Carlo (MCMC) approaches [15, 16].

Despite the value of individual plasma drug concentration-time profiles, which can be analysed using NLME modeling, such detailed data are often unavailable, especially in older or non-modeling-focused studies. Instead, a common practice in the literature is to report only summary statistics, typically the mean and standard deviation of the concentration measurements at each time point. These are often visualized using mean values as points and standard deviations as error bars. While this format is widespread, it provides only a limited view of the underlying pharmacokinetic variability, and estimation methods are needed to extract as much information as possible from these data. Full Bayesian MCMC approaches have previously been proposed to analyze reported concentration means and variances in a meta-analysis context [17–19].

**Fig. 1:**
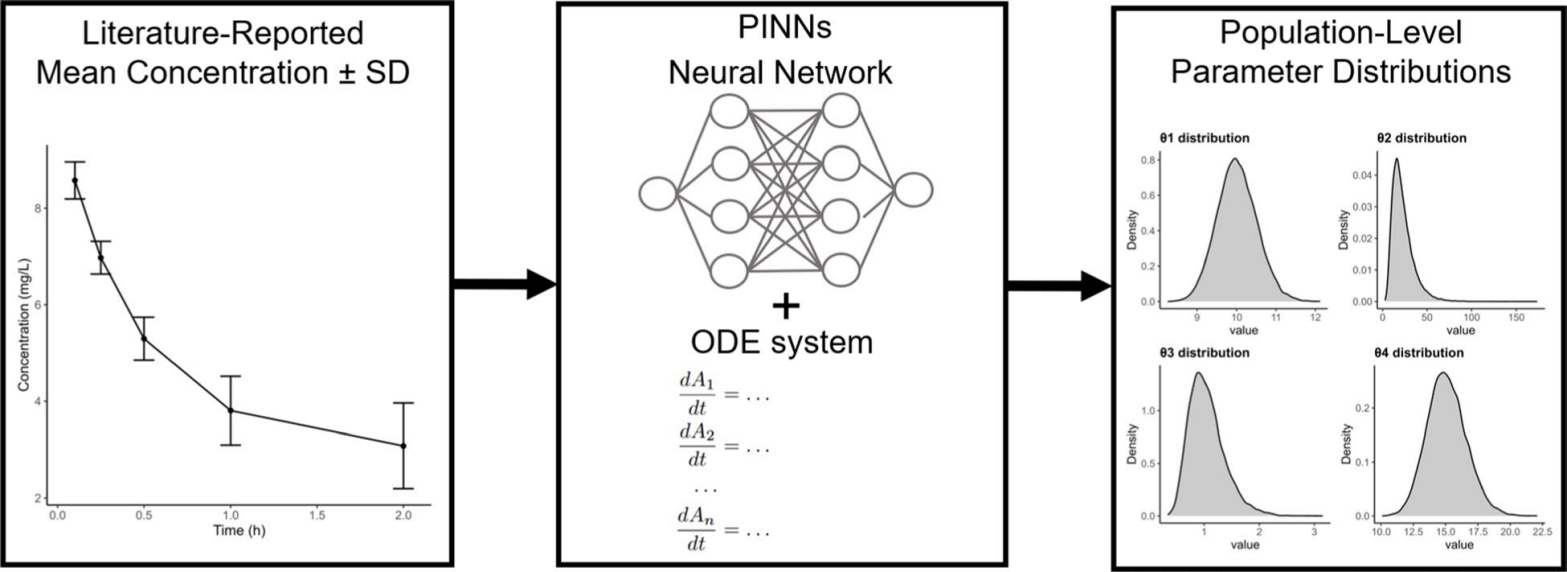
Simplified representation of the D-PINNs framework. A physics-informed neural network (PINN), combining a neural network with an ODE system describing the pharmacokinetic model, extracts information from aggregated concentration data. Through this process, the population-level distributions of the system parameters are inferred

In this work, we introduce a variant of PINNs, termed distributional PINNs (D-PINNs), designed to estimate the underlying parameters of a pharmacokinetic system of ordinary differential equations (ODEs) using only the reported mean and variance of concentration data over time. This framework allows for the recovery of parameter distributions that produce the observed concentration distributions, rather than merely providing point estimates. A simplified schematic representation of the proposed method is illustrated in Fig. 1.

## Methods

### D-PINNS: distributional physics-informed neural networks

Consider a pharmacokinetic (PK) system described by a system of ODEs of the form:

1 $$\begin{aligned} \begin{aligned} \dfrac{d\boldsymbol{C}(t)}{dt} = \boldsymbol{f}(\boldsymbol{C}(t), t, \boldsymbol{\eta }), \quad \boldsymbol{C}(0) = \boldsymbol{C}_0 \end{aligned} \end{aligned}$$

where $\boldsymbol{C}(t) \in \mathbb {R}^n$ denotes the vector of drug concentrations across $N_c$ compartments, $\boldsymbol{f}(\cdot )$ denotes the right-hand side of the ODE system, defining the system dynamics, $\boldsymbol{C}_0$ specifies the initial condition, and $\boldsymbol{\eta }$ represents the parameters of the ODE system. The solution to this system can be approximated by a neural network $\widehat{\boldsymbol{C}}(t)$, which takes time $t \in [0, T]$ as input and produces estimated concentration values. Here, $\boldsymbol{\theta _{\text {NN}}}$ denotes the trainable parameters of the neural network, i.e., its weights and biases.

The key idea of PINNs is to incorporate knowledge of the system’s dynamics into the loss function through the residual of the ODE system, defined as:

2 $$\begin{aligned} \begin{aligned} \boldsymbol{\mathcal {R}}(t, \boldsymbol{\eta }) = \dfrac{d\widehat{\boldsymbol{C}}(t)}{dt} - \boldsymbol{f}(\widehat{\boldsymbol{C}}(t), t, \boldsymbol{\eta }) \end{aligned} \end{aligned}$$

where $\frac{d}{dt}$ denotes the time derivative of the neural network output. Although various numerical schemes are possible, PINNs typically use automatic differentiation to compute this derivative because it provides both accuracy and numerical stability [20]. The total loss function used to train the PINN is composed of three terms:

3 $$\begin{aligned} \begin{aligned} \mathcal {L}_{\text {total}}= \lambda _{\text {ODE}} \mathcal {L}_{\text {ODE}} + \lambda _{\text {IC}} \mathcal {L}_{\text {IC}} + \lambda _{\text {D}} \mathcal {L}_{\text {D}} \end{aligned} \end{aligned}$$

where $\mathcal {L}_{\text {ODE}}$, $\mathcal {L}_{\text {IC}}$, and $\mathcal {L}_{\text {D}}$ are the ODE, initial condition and data loss terms, and $\lambda _{\text {ODE}}$, $\lambda _{\text {IC}}$, and $\lambda _{\text {D}}$ are the corresponding loss weights. The individual loss terms are defined as:

4 $$\begin{aligned} \begin{aligned} \mathcal {L}_{\text {ODE}} = \dfrac{1}{|N_{cp}|} \sum _{j=1}^{N_{cp}} \left\| \left. \dfrac{d\widehat{\boldsymbol{C}}(t_j)}{dt} \right| _{\text {AD}} - \boldsymbol{f}(\widehat{\boldsymbol{C}}(t_j), t_j, \boldsymbol{\eta }) \right\| _2^2 \end{aligned} \end{aligned}$$

5 $$\begin{aligned} \begin{aligned} \mathcal {L}_{\text {IC}}= \dfrac{1}{|N_c|} \left\| \widehat{\boldsymbol{C}}(0) - \boldsymbol{C}_0 \right\| _2^2 \end{aligned} \end{aligned}$$

6 $$\begin{aligned} \begin{aligned} \mathcal {L}_{\text {D}}= \dfrac{1}{|N_{\text {obs}}|} \sum _{j=1}^{N_{\text {obs}}} \left\| \widehat{\boldsymbol{C}}(t_j) - \boldsymbol{C}(t_j) \right\| _2^2 \end{aligned} \end{aligned}$$

where $N_{cp}$ denotes the collocation points at which the residuals are evaluated, $N_c$ reprents the number of compartments, $N_{\text {obs}}$ corresponds to the number of observations, and $\left. \frac{d}{dt}\right| _{\text {AD}}$ indicates that the derivative is computed via automatic differentiation of the neural network. The training process involves jointly optimising the neural network parameters $\boldsymbol{\theta }_{\text {NN}}$ and the ODE system parameters $\boldsymbol{\eta }$ by minimising a composite loss function that integrates both the system dynamics and the observed data.

**Fig. 2:**
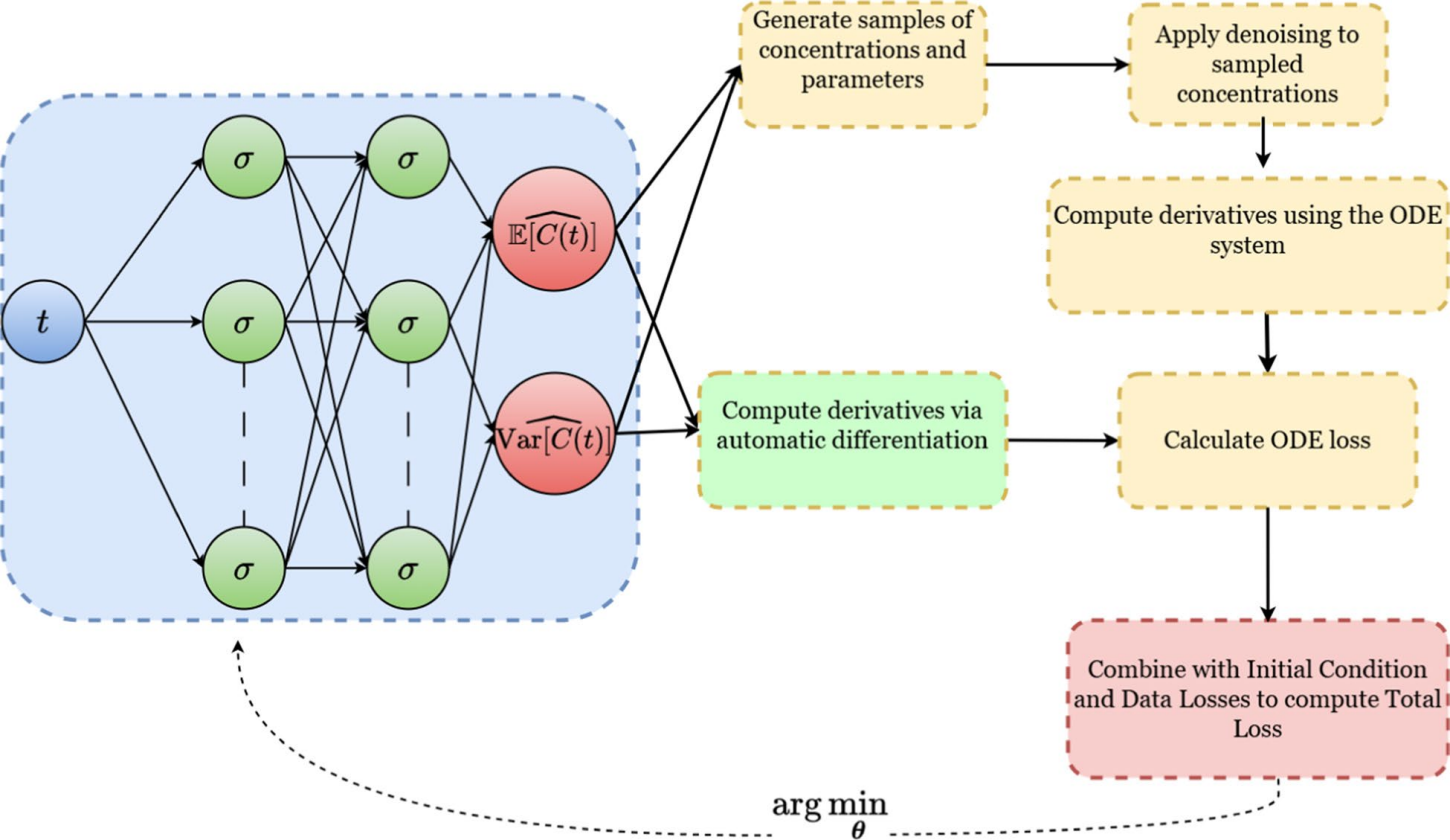
Schematic representation of the D-PINN algorithm applied to a simple one-dimensional problem. This schematic highlights the core component of the algorithm: the computation of the ODE loss term. The method integrates a neural network (NN) with a system of ordinary differential equations (ODEs). At its core, the NN outputs the estimated mean and variance of the concentration over time, which are used to generate noisy concentration samples. These samples are then denoised and passed through the ODE system to compute the time derivatives of the mean and variance. These are directly compared to the corresponding derivatives from automatic differentiation of the neural network to calculate the ODE loss

The core principle of D-PINNs lies in incorporating both the mean and variance of the system’s state into the optimisation process by adopting appropriate distributional assumptions. In this framework, the neural network does not predict concentration values directly, but instead outputs the mean and variance of the concentration, which serve as statistical summaries of the observed data. Consequently, each concentration value, previously modelled as a single deterministic output, is now represented by two outputs: one for the predicted mean and one for the predicted variance. These predictions are used inside a sampling framework that employs the ODE system to compute the ODE loss term. Figure 2 presents a simplified schematic of the D-PINN framework, offering a high-level view of its key components prior to the detailed formulation.

Building on the schematic representation, we now present the full mathematical formulation of the D-PINN framework. This extension of the original PINN formulation introduces a total loss function that simultaneously accounts for the mean and variance of the predicted system state:

7 $$\begin{aligned} \begin{aligned} \mathcal {L}_{\text {total}} &= \lambda _{\text {ODE}}^{\mathbb {E}} \mathcal {L}_{\text {ODE}}^{\mathbb {E}} + \lambda _{\text {ODE}}^{\textrm{Var}} \mathcal {L}_{\text {ODE}}^{\textrm{Var}} + \lambda _{\text {IC}}^{\mathbb {E}} \mathcal {L}_{\text {IC}}^{\mathbb {E}} \\&\quad+ \lambda _{\text {IC}}^{\textrm{Var}} \mathcal {L}_{\text {IC}}^{\textrm{Var}} + \lambda _{\text {D}}^{\mathbb {E}} \mathcal {L}_{\text {D}}^{\mathbb {E}} + \lambda _{\text {D}}^{\textrm{Var}} \mathcal {L}_{\text {D}}^{\textrm{Var}} \end{aligned} \end{aligned}$$

with:

8 $$\begin{aligned} \begin{aligned} \mathcal {L}_{\text {ODE}}^{\mathbb {E}}= \dfrac{1}{|N_{cp}|} \sum _{j=1}^{N_{cp}} \left\| \left. \dfrac{d\,\widehat{\mathbb {E}[\boldsymbol{C}(t_j)]}}{dt} \right| _{\text {AD}} - \left. \dfrac{d\,\mathbb {E}[\boldsymbol{C}(t_j)]}{dt} \right| _{\text {ODE}} \right\| _2^2 \end{aligned} \end{aligned}$$

9 $$\begin{aligned} \begin{aligned} \mathcal {L}_{\text {ODE}}^{\textrm{Var}} = \dfrac{1}{|N_{cp}|} \sum _{j=1}^{N_{cp}} \left\| \left. \dfrac{d\widehat{\textrm{Var}[\boldsymbol{C}(t_j)]}}{dt} \right| _{\text {AD}} - \left. \dfrac{d{\textrm{Var}[\boldsymbol{C}(t_j)]}}{dt} \right| _{\text {ODE}} \right\| _2^2 \end{aligned} \end{aligned}$$

10 $$\begin{aligned} \begin{aligned} \mathcal {L}_{\text {IC}}^{\mathbb {E}} = \dfrac{1}{|N_c|} \left\| \widehat{\mathbb {E}[\boldsymbol{C}(t_0)]} - \mathbb {E}[\boldsymbol{C}_{0}(\boldsymbol{\eta })] \right\| _2^2 \end{aligned} \end{aligned}$$

11 $$\begin{aligned} \begin{aligned} \mathcal {L}_{\text {IC}}^{\textrm{Var}}= \dfrac{1}{|N_c|} \left\| \widehat{\textrm{Var}[\boldsymbol{C}(t_0)]} - \textrm{Var}[\boldsymbol{C}_0(\boldsymbol{\eta })] \right\| _2^2 \end{aligned} \end{aligned}$$

12 $$\begin{aligned} \begin{aligned} \mathcal {L}_{\text {D}}^{\mathbb {E}} = \dfrac{1}{|N_{\text {obs}}|} \sum _{j=1}^{N_{\text {obs}}} \left\| \widehat{\mathbb {E}[\boldsymbol{C}(t_j)]} - \mathbb {E}[\boldsymbol{C}(t_j)] \right\| _2^2 \end{aligned} \end{aligned}$$

13 $$\begin{aligned} \begin{aligned} \mathcal {L}_{\text {D}}^{\textrm{Var}} = \dfrac{1}{|N_{\text {obs}}|} \sum _{j=1}^{N_{\text {obs}}} \left\| \widehat{\textrm{Var}[\boldsymbol{C}(t_j)]} - \textrm{Var}[\boldsymbol{C}(t_j)] \right\| _2^2 \end{aligned} \end{aligned}$$

where $\widehat{\mathbb {E}[\boldsymbol{C}(t_j)]}$ and $\widehat{\textrm{Var}[\boldsymbol{C}(t_j)]}$ denote the mean and variance of the concentration at time point $t_j$ predicted by the NN, while $\mathbb {E}[\boldsymbol{C}(t_j)]$ and $\textrm{Var}[\boldsymbol{C}(t_j)]$ correspond to the observed mean and variance of the concentration at time point $t_j$. The notation $\left. \frac{d}{dt}\right| _{\text {ODE}}$ indicates that the derivative is estimated directly through the ODE system using sampling.

We assume a single homogeneous population from which individual-specific parameters are drawn. In this setting, the population-level variability corresponds directly to interindividual variability, reflecting variability among individual subjects around a single common population mean. The aim of D-PINNs is to estimate the population distribution of the ODE parameters $\boldsymbol{\eta }$, described by distributional parameters $\boldsymbol{\theta }_{\eta }$, based on estimates of the concentration mean and variance. The model accounts for interindividual variability explicitly through the distribution of $\boldsymbol{\eta }$. Residual variability is assumed to originate solely from measurement error, including sources such as assay variability and sample handling error. As a result, the observed concentrations reflect both interindividual variability and measurement error. Accordingly, the associated sample means and variances are noisy estimates of the true underlying moments. Assuming a log-normal error model, the noise-free concentration at each time point, denoted by $\boldsymbol{C}^{\text {true}}(t)$, can be related to the observed concentration $\boldsymbol{C}(t)$ via:

14 $$\begin{aligned} \boldsymbol{C}(t)&= \boldsymbol{C}^{\text {true}}(t) \cdot e^{\epsilon } , \quad \boldsymbol{\epsilon } \sim \mathcal {N}(0, \sigma ^2) \end{aligned}$$

where $\boldsymbol{\epsilon }$ is the residual error on the log scale, representing measurement error, and $\sigma$ is the residual standard deviation on the log scale. Parameter $\sigma$ is treated as a trainable parameter in this framework. The distinction between $\boldsymbol{C}^{\text {true}}(t)$ and $\boldsymbol{C}(t)$ is critical, as it allows the model to account for residual variability and disentangle noisy observations from the underlying true concentrations that are used in the ODE system, as will be elaborated later in this section.

At each iteration, a forward pass with the current estimate of $\boldsymbol{\theta }_{\text {NN}}$ yields updated predictions for the noisy concentration mean and variance, $\widehat{\mathbb {E}[\boldsymbol{C}(t)]}$ and $\widehat{\textrm{Var}[\boldsymbol{C}(t)]}$, respectively. These predictions, along with the updated distributional parameter estimates $\boldsymbol{\theta }_{\eta }$, are appropriately transformed to parameterise a predefined joint distribution, $p( \boldsymbol{C}(t), \boldsymbol{\eta })$, from which samples of $\boldsymbol{C}(t)$ and $\boldsymbol{\eta }$ are drawn. Residual errors are sampled using the current estimate of the residual standard deviation $\sigma$ and are applied to Eq. 14 to correct for measurement noise. This denoising step yields sampled estimates of the true concentration, $\boldsymbol{C}^{\text {true}}(t)$. For each sampled instance (*i*), the corresponding denoised concentration $\boldsymbol{C}^{\text {true}, (i)}(t)$ and parameter vector $\boldsymbol{\eta }^{(i)}$ are subsequently used to compute the respective time derivative:

15 $$\begin{aligned} \begin{aligned} \dfrac{d\boldsymbol{C}^{\text {true},(i)}(t)}{dt} = \boldsymbol{f}(\boldsymbol{C}^{\text {true}, (i)}(t), t, \boldsymbol{\eta }^{(i)}) \end{aligned} \end{aligned}$$

The resulting distribution of derivatives is then used to compute the ODE-based estimates of the time derivatives of the denoised concentration mean and variance, as follows:

16 $$\begin{aligned} \begin{aligned} \left. \dfrac{d\,\mathbb {E}[\boldsymbol{C}^{\text {true}}(t)]}{dt} \right| _{\text {ODE}}&= \dfrac{1}{|N_{\text {samples}}|} \sum _{i=1}^{N_{\text {samples}}} \dfrac{d\,\boldsymbol{C}^{\text {true},(i)}(t)}{dt} \end{aligned} \end{aligned}$$

17 $$\begin{aligned} \begin{aligned} \left. \dfrac{d\,\textrm{Var}[\boldsymbol{C^{\text {true}}}(t)]}{dt} \right| _{\text {ODE}}&= \dfrac{2}{|N_{\text {samples}}| - 1} \sum _{i=1}^{N_{\text {samples}}} \Bigg [ \left( C^{\text {true}, (i)}(t) - \mathbb {E}[\boldsymbol{C}^{\text {true}}(t)] \right) \cdot \\&\quad \hspace{5em}\left( \dfrac{d\,\boldsymbol{C}^{\text {true},(i)}(t)}{dt} - \left. \dfrac{d\,\mathbb {E}[\boldsymbol{C}^{\text {true}}(t)]}{dt} \right| _{\text {ODE}} \right) \Bigg ] \end{aligned} \end{aligned}$$

where $N_{\text {samples}}$ represents the total number of drawn samples. A full derivation of these expressions, along with the associated assumptions, is provided in Appendix A. The final step consists of reconstructing the ODE-based derivatives of the observed concentration mean and variance from the denoised estimates via the following transformation:

18 $$\begin{aligned} \left. \frac{d \mathbb {E}[C(t)]}{dt}\right| _{\text {ODE}}&= e^{{\sigma ^2}/2} \cdot \left. \frac{d\mathbb {E}[C^{\text {true}}(t)]}{dt} \right| _{\text {ODE}} \end{aligned}$$

19 $$\begin{aligned} \left. \frac{d\textrm{Var}[C(t)]}{dt} \right| _{\text {ODE}}&= e^{\sigma ^2} \cdot \left[ 2 \cdot \mathbb {E}[C^{\text {true}}(t)] \cdot \left. \frac{d\mathbb {E}[C^{\text {true}}(t)]}{dt} \right| _{\text {ODE}} \cdot \left( e^{\sigma ^2} - 1 \right) \right. \nonumber \\&\quad \left. + e^{\sigma ^2} \cdot \left. \frac{d\textrm{Var}[C^{\text {true}}(t)]}{dt} \right| _{\text {ODE}} \right] \end{aligned}$$

The derivation of Eqs. 18 and 19 is provided in Appendix B. This parameterisation allows the model to simultaneously estimate both the population-level distribution of the ODE parameters and the measurement error in the observed concentrations. The ODE-based estimates of the observed mean and variance derivatives are then compared against those obtained via automatic differentiation of the NN, through Eqs. 8 and 9, to compute the ODE-related loss terms. When the system’s initial conditions depend on the ODE parameters $\boldsymbol{\eta }$, the same sampling workflow is applied to estimate $\mathbb {E}[\boldsymbol{C}_0(\boldsymbol{\eta })]$ and $\textrm{Var}[\boldsymbol{C}_0(\boldsymbol{\eta })]$. The trainable parameters of the system, namely the neural network parameters $\boldsymbol{\theta }_{\text {NN}}$, the distributional parameters $\boldsymbol{\theta }_{\eta }$, and the residual standard deviation $\sigma$, collectively denoted by $\boldsymbol{\theta }$, are optimised by minimising the total loss function. Algorithm 1 outlines the computational workflow of the D-PINNs framework, while Fig. 2 illustrates its core components in the context of a simple one-compartment ODE system.

**Algorithm 1:**
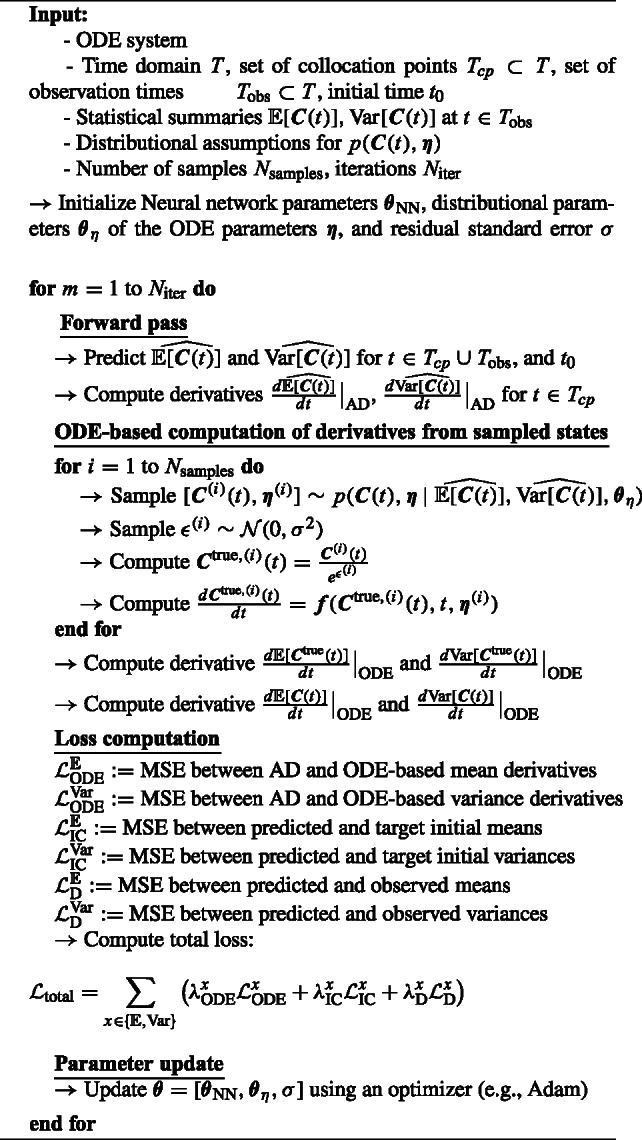
D-PINNs Algorithm

### Case study 1: simulated data from a one-compartment pharmacokinetic model

**Fig. 3:**
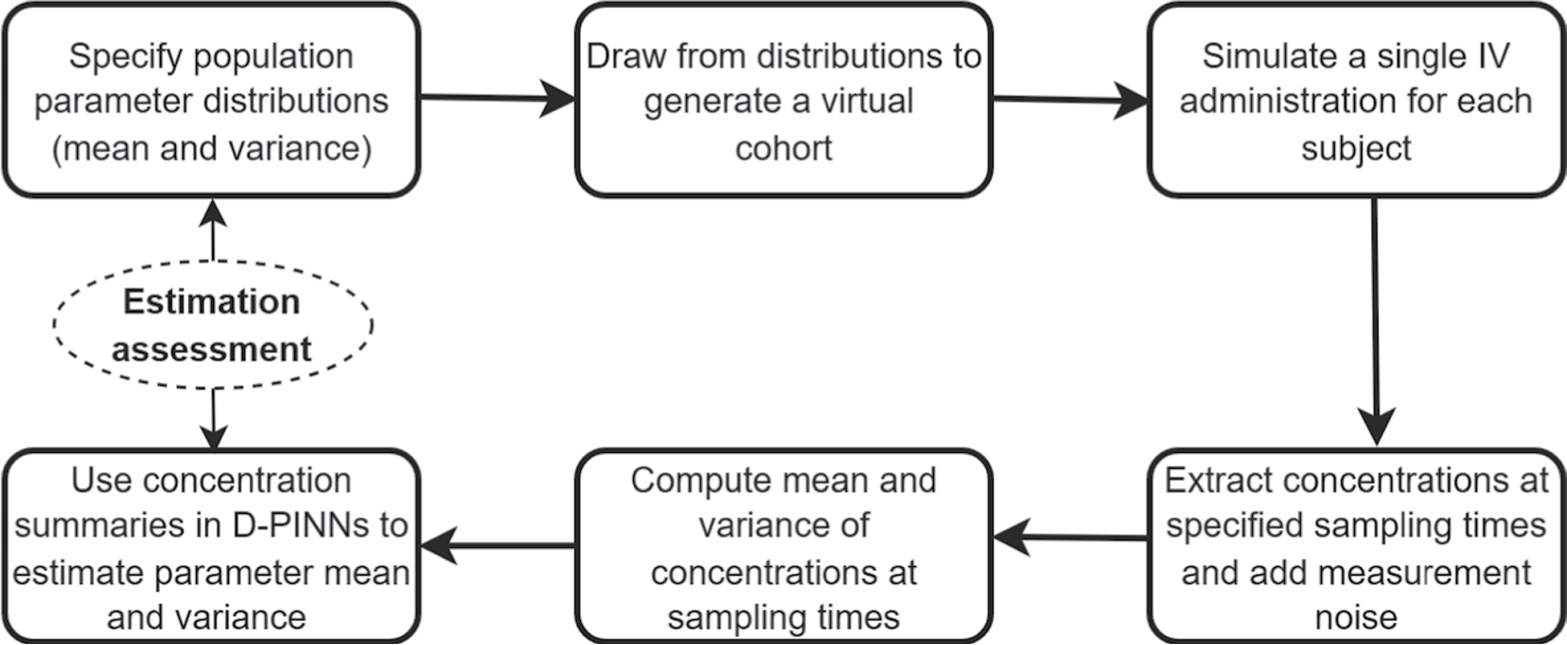
Modelling workflow for Case Study 1. A predefined population parameter distribution is used to generate a virtual cohort and simulate IV concentration–time profiles. After adding measurement noise and sampling at predefined time points, the concentration mean and variance are computed. These summary statistics are then provided as training data to the D-PINN model, which estimates the mean and variance of each population parameter. The estimated distributions are finally compared to the ground-truth distributions used to generate the virtual cohort

The aim of case study 1 is to illustrate the application of D-PINNs for estimating population-level pharmacokinetic parameters. To showcase the capabilities of the methodology, we selected a simple one-compartment model with intravenous (IV) bolus dosing as a representative system. This model was chosen for its simplicity, widespread use in early-phase pharmacokinetic studies, and its suitability as a tractable framework for evaluating new inference approaches. Simulated data were generated using predefined population-level pharmacokinetic parameters. The performance of the D-PINN framework was then assessed by comparing the estimated parameters to the reference values used during simulation. The modelling workflow of case study 1 is illustrated in Fig. 3.

#### Pharmacokinetic system

**Fig. 4:**
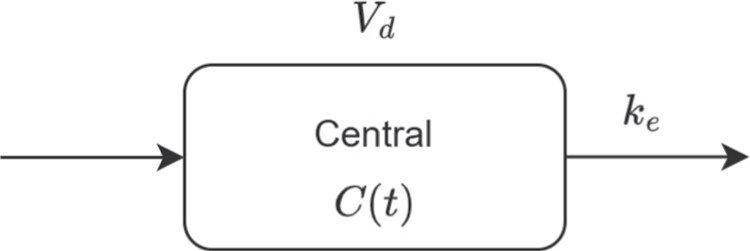
Schematic presentation of the simple, one-compartment pharmacokinetic model

The one-compartment pharmacokinetic model describes a simplified system in which the drug is assumed to distribute instantaneously and uniformly within a central compartment. Following a bolus IV injection, the drug enters directly into the central compartment. The model is schematically presented in Fig. 4.

The ODE system that expresses the pharmacokinetic model is presented in Eqs. 20 - 21. It consists of a single state describing the concentration evolution of the drug in plasma over time, *C*(*t*). The pharmacokinetics of the system are characterized by two parameters: the volume of distribution ($V_d$), which governs how extensively the drug partitions between plasma and peripheral tissues, and the elimination rate constant ($k_e$), which governs the rate at which the drug is removed from the system. At time zero, the initial concentration of the drug was set to the total dose divided by $V_d$.

20 $$\begin{aligned} \dfrac{dC(t)}{dt}&= - k_{e} C(t) \end{aligned}$$

21 $$\begin{aligned} C(t_0)&= \frac{\text {Dose}}{V_d} \end{aligned}$$

#### Simulated data

The presented one-compartment model was used to simulate a cohort of 30 virtual patients, each characterized by individual pharmacokinetic parameters. These parameters were sampled from a multivariate population distribution and served as inputs to the ODE system to generate patient-specific plasma concentration-time profiles. Specifically, $V_d$ and $k_e$ were jointly sampled from a multivariate log-normal distribution. The population mean for $V_d$ was set to $15 \, \text {L}$, with a coefficient of variation (CV) of $10\%$. The mean of $k_e$ was defined by specifying a mean half-life in blood equal to $6 \, \text {h}$ ($k_e \approx 0.12$), and its standard deviation was defined through a CV of $40\%$. The correlation between $V_d$ and $k_e$ was varied to explore its impact on the inference process.

In the simulated scenario, a $300 \, \text {mg}$ bolus intravenous dose was assumed, and plasma concentrations were computed at 0.5, 1, 2, 6, 12, and 24 h post-administration, reflecting a clinically relevant sampling schedule. To account for residual variability, a log-normal error model was used, with log-scale standard deviation set to $\sigma = 0.1$. Thus, for each virtual patient *k* and observation time point $t_j$, the observed concentration was generated using the following model:

22 $$\begin{aligned} \log \boldsymbol{\eta }_k&= [\log V_{d_k}, \log k_{e_k}] \sim \textrm{MVN}(\boldsymbol{\mu }, \Omega ) \end{aligned}$$

23 $$\begin{aligned} C_{k}(t_j)&= C^{\text {true}}_{k}(t_j) \cdot e^{\epsilon _{kj}}, \quad \epsilon _{kj} \sim \mathcal {N}(0, \sigma ^2) \end{aligned}$$

where $\textrm{MVN}$ denotes the multivariate normal distribution, $\boldsymbol{\mu }$ and $\Omega$ represent the mean vector and covariance matrix, and $\epsilon _{kj}$ denotes the corresponding measurement error. The distributional parameters are thus defined as $\boldsymbol{\theta }_{\eta } = [\boldsymbol{\mu }, \Omega ]$. The resulting data were summarized by computing the mean and variance at each time point and subsequently used as input for the D-PINN analysis. Therefore, the reported summary statistics at each time point reflect both interindividual pharmacokinetic variability and measurement noise.

#### D-PINN implementation

The objective of the case study is to demonstrate that, under the true structural and statistical models, D-PINNs can recover the pharmacokinetic parameter distribution using only aggregated plasma concentration data. Accordingly, the same ODE system was employed, and the D-PINN was configured to assume the same probability distribution for the system parameters as the one used during data simulation. At each iteration, the mean and variance estimates of the NN at time points $t_j \in \{t_0\} \cup T_{cp}$, $\widehat{\mathbb {E}[\boldsymbol{C}(t)]}$ and $\widehat{\textrm{Var}[\boldsymbol{C}(t_j)]}$, were used alongside the distributional parameters $\boldsymbol{\theta _{\eta }}$ to parameterise a joint probability distribution. Specifically, for each time point $t_j$, $j = 1, \cdots , N_{cp}$, and sample index $i = 1, 2, \dots , N_{\text {samples}}$, draws from the joint distribution over plasma concentration and ODE parameters were obtained as follows:

24 $$\begin{aligned} {[}\log C^{(i)}(t_j), \log V_{d_j}^{(i)}, \log k_{e_j}^{(i)}{]} \sim \textrm{MVN}(\boldsymbol{\mu }\boldsymbol{'}_{\!\!\boldsymbol{j}}, \Omega _j') \end{aligned}$$

where $\boldsymbol{\mu _j}', \Omega _j'$ were constructed by first transforming $\widehat{\mathbb {E}[\boldsymbol{C}(t_j)]}$ and $\widehat{\textrm{Var}[\boldsymbol{C}(t_j)]}$ into their corresponding log-scale parameters by applying standard transformations from linear-scale moments to log-scale parameters of the log-normal distribution. The transformed estimates were then combined with the already log-transformed distributional parameters $\boldsymbol{\theta }_{\eta }$ to form $\boldsymbol{\mu _j}'$ and $\Omega _j'$ . While the mean and variance estimates are time-dependent, distributional parameters $\boldsymbol{\theta }_{\eta }$ remain fixed across time. Nevertheless, samples of $V_d$ and $k_e$ were drawn across time to better capture the range of parameters capable of reproducing the observed plasma statistical summaries. Additionally, to enable joint sampling of the ODE parameters and plasma concentration, the covariance matrix $\Omega _j'$ also included the correlations between plasma concentration and ODE parameters. These correlation terms were assumed time-invariant and were treated as trainable parameters. Finally, sampled noisy concentrations were denoised using Eq. 14, and then mean and variance ODE-based time derivatives were computed by applying Eqs. 16 - 19.

In addition to the ODE-based loss, samples of $V_d$ were also used to compute the initial condition loss terms. The neural network outputs the mean and variance of the observed concentrations, which reflect both biological variability and measurement noise. This should not be confused with the typical ODE initial conditions, which account only for biological variability. Consequently, the initial condition of the network must incorporate the effects of measurement noise. Specifically, samples of $V_d$ at time $t_0$ were used to estimate the observed initial concentration as:

25 $$\begin{aligned} C^{(i)}(t_0) = \frac{\text {Dose}}{V_d^{(i)}} \cdot e^{\epsilon } , \quad \epsilon \sim \mathcal {N}(0, \sigma ^2) \end{aligned}$$

These samples were then summarised to compute the mean and variance of the initial observed concentration. The resulting statistical summaries were incorporated into Eqs. 10 and 11, along with NN estimates $\widehat{\mathbb {E}[C](t_0)}$ and $\widehat{\textrm{Var}[C(t_0)]}$, to derive the initial condition loss.

Regarding implementation details, a simple feedforward NN was employed, consisting of two hidden layers with three nodes each. The *tanh* activation function was applied to all layers, while the output layer included the *softplus* transformation to ensure non-negative NN outputs. NN weights were initialised using the Glorot normal initialiser, and no loss regularisation was applied. The model was trained using the Adam optimizer with a learning rate of 0.0005. To further enhance model stability, gradient clipping was performed to constrain the Euclidean norm of all gradients to be less than 10. The impact of the number of samples $N_{\text {samples}}$, the number of collocation points $N_{cp}$ and the choice of loss weights on predictive performance was assessed using simulations. All trainable parameters representing elements of the correlation matrix parameters were transformed using the *tanh* function to constrain their values within the [-1,1] interval. Similarly, all trainable parameters representing standard deviations were transformed using the *softplus* function with a small positive bias, to ensure strictly positive values.

Finally, the accuracy of each individual distributional parameter estimate $\theta _{\eta _p}^{\text {estimated}}$ was compared to the true parameter $\theta _{\eta _p}^{true}$ using the absolute percentage error (APE), as defined in Eq. 26. The overall estimation accuracy across all $N_{\text {par}}$ distributional parameters was evaluated using the mean absolute percentage error (MAPE), shown in Eq. 27.

26 $$\begin{aligned} \textrm{APE}_p&=100\%\times \left| \frac{\theta _{\eta _p}^{true}-\theta _{\eta _p}^{estimated}}{\theta _{\eta _p}^{true}}\right| , \quad p=1,\dots ,N_{\text {par}} \end{aligned}$$

27 $$\begin{aligned} \textrm{MAPE}&=\frac{1}{N_{\text {par}}}\sum _{p=1}^{N_{par}}\textrm{APE}_p \end{aligned}$$

### Case study 2: parameter estimation for a minimal physiologically-based pharmacokinetic model

The main goal of case study 2 is to employ D-PINNs in a real-world pharmacokinetic modeling estimation problem. For this purpose, a minimal physiologically based pharmacokinetic (mPBPK) model was implemented to simulate the kinetics of TAB008 (later marketed as Pusintin), a biosimilar monoclonal antibody (mAb) of bevacizumab (Avastin). The aggregated mAb plasma concentrations were also modelled using a Bayesian analogue implemented using the Markov chain Monte Carlo (MCMC) methodology to benchmark the speed and estimation performance of D-PINNs .

#### Monoclonal antibody data

The aggregated concentration data were drawn from a phase I clinical study of TAB008 [21]. The pharmacokinetic study included 49 healthy Chinese male subjects aged 18-45 years old, with body-weights ranging from 50 to 75 kg. Each subject received a single IV dose of 1 mg/kg of TAB008, administered over a 90-minute infusion. A sample was collected at 0.75 hours after the start of infusion, followed by five additional samples between 0 and 8 hours post-infusion. Sampling was further extended up to 99 days post administration to study long term kinetics. The online software PlotDigitizer [22] was used to extract the reported mean and standard deviation of serum concentrations over time from the published figure. In total, 15 measurements of aggregated plasma concentrations over time were extracted from the figure and used to estimate the pharmacokinetic parameters of the mAb.

#### Minimal physiologically-based pharmacokinetic model

**Fig. 5:**
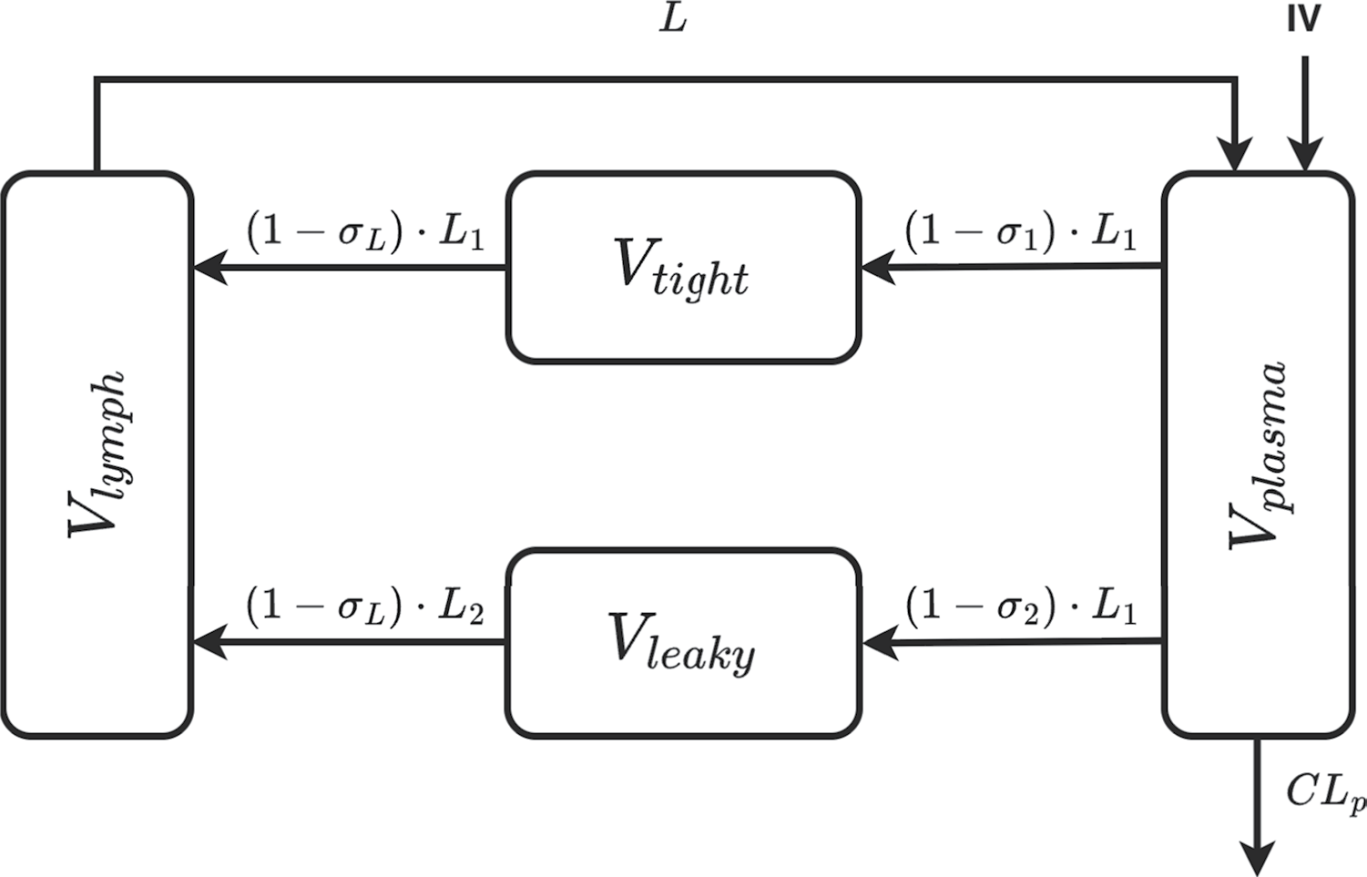
Structural representation of the mPBPK model, redrawn and adapted from [23]. The current implementation considers that mAbs are eliminated only through the plasma compartment

The implemented model follows a minimal modelling approach specifically designed to characterise mAb kinetics [23]. It assumes that convection is the primary mechanism driving mAb distribution into the extravascular space. Because mAbs exhibit limited permeability across cellular membranes, the interstitial fluid (ISF) compartment is considered the main extravascular space for distribution. Accordingly, tissues are grouped into two categories based on capillary structure: continuous or discontinuous. The discontinuous (leaky) group includes tissues such as the liver, kidney, and heart, whereas the continuous (tight) group includes muscle, skin, and adipose tissue. Transport of mAbs from plasma to ISF is governed by tissue-specific reflection coefficients, and lymphatic return to plasma is similarly regulated by a distinct reflection coefficient. A structural representation of the mPBPK model is shown in Fig. 5.

The differential equations that describe the dynamics of the system follow the formulation of Cao et al. [23] and are provided below:

28 $$\begin{aligned}&\frac{dC_{plasma}}{dt} = \left[ \frac{Dose}{T_{inf}} +C_{lymph} \cdot L - C_{plasma} \cdot L_1 \cdot (1 - \sigma _1) \right. \nonumber \\&\qquad \qquad \left. - C_{plasma} \cdot L_2 \cdot (1 - \sigma _2) - C_{plasma}\cdot CL_p\right] /V_{plasma} \end{aligned}$$

29 $$\begin{aligned}\frac{dC_{tight}}{dt} &= \left[ L_1 \cdot (1 - \sigma _1) \cdot C_{plasma} \right.\\&\left.\quad- L_1 \cdot (1 - \sigma _L) \cdot C_{tight}\right] /V_{tight} \end{aligned}$$

30 $$\begin{aligned}\frac{dC_{leaky}}{dt} &= \left[ L_2 \cdot (1 - \sigma _2) \cdot C_{plasma} \right.\\&\quad\left.- L_2 \cdot (1 - \sigma _L) \cdot C_{leaky}\right] /V_{leaky} \end{aligned}$$

31 $$\begin{aligned}\frac{dC_{lymph}}{dt}&= \Bigg[ L_1 \cdot (1 - \sigma _L) \cdot C_{tight} + L_2 \cdot (1 - \sigma _L) \\& \cdot C_{leaky} - C_{lymph}\cdot L \Bigg] /V_{lymph} \end{aligned}$$

In Eqs. 28 to 31, $C_{plasma}$ denotes the plasma concentration, $C_{lymph}$ is the lymph concentration, and $C_{tight}$ and $C_{leaky}$ are the concentrations in the tight and leaky and compartments, respectively. The term $T_{inf}$ represents the IV infusion time, $V_{plasma}$ and $V_{lymph}$ denote the plasma and lymph volumes, and $V_{tight}$ and $V_{leaky}$ correspond to the total ISF volumes in tight and leaky tissues. Moreover, *L* represents the total lymph flow, while $L_1$ and $L_2$ represent the lymph flow to tight and leaky compartments. Finally, $CL_p$ denotes the clearance rate from plasma, $\sigma _L$ is the lymphatic capillary reflection, and $\sigma _1$ and $\sigma _2$ represent the vascular reflection coefficients for the tight and leaky compartments, respectively. A detailed parameterisation of the model is provided in Appendix C.

The model imposes several physical constraints on the parameters. Specifically, both $\sigma _1$ and $\sigma _2$ must be lower than 1, since the extravasation rate cannot be higher than the total lymph flow (*L*). In addition, they must satisfy the inequality $\sigma _1> \sigma _2$, since tight vascular endothelium tissues have a higher reflection than leaky tissues.

#### D-PINN implementation

The aggregated plasma concentration data were used to estimate plasma clearance, $CL_p$, and the reflection coefficients of tight and leaky tissues, $\sigma _1$ and $\sigma _2$, respectively. Among these parameters, only $CL_p$ was treated as a stochastic parameter to account for interindividual variability within the studied population. In contrast, point estimates were used for the reflection coefficients. This choice is supported by the physiological nature of the reflection coefficients, which characterise permeability through the capillary membranes, and are not expected to exhibit high interindividual variability. Apart from interindividual variability in clearance, the D-PINN implementation also incorporated variability in physiological parameters by sampling the body weight of virtual subjects from a uniform distribution defined by the minimum and maximum values (50 and 75 kg) reported in [21]. All physiological parameters dependent on body weight ($V_{blood}, V_{lymph}, V_{plasma}, V_{ISF}, L$, $L_1$ and $L_2$) were adjusted accordingly (see Eqs. 65 to 72 in Appendix C). Finally, the administered dose was scaled based on the sampled body weight, according to the 1 mg/kg dosing regimen followed in the trial [21].

The output layer of the NN was adjusted to the availability of aggregated concentration data. Specifically, two nodes were used for the aggregated plasma concentration, representing the central tendency and spread of the plasma concentration, and a single output node was employed for each unobserved state, including the concentration in the lymph, tight and leaky tissues. The observed variance in the clinical data spanned a wide range (0.01–9), covering almost three orders of magnitude. To better scale this wide range and avoid the optimisation issues associated with large output disparities, we modelled the standard deviation instead of the variance. Data loss terms were linked only to the first two output nodes, while all initial condition loss terms were set to zero. The ODE-based derivative of the ODE loss term for the standard deviation was estimated using the following equation:

32 $$\begin{aligned} \left. \frac{d\,\textrm{SD}[C(t)]}{dt} \right| _{\text {ODE}}&= \frac{\left. \frac{d\,\textrm{Var}[C(t)]}{dt} \right| _{\text {ODE}}}{2\cdot \widehat{\textrm{SD}[C(t)]}} \end{aligned}$$

where $\widehat{\textrm{SD}[\boldsymbol{C}(t)]}$ is the standard deviation predicted by the NN and $\left. \frac{d\,\textrm{Var}[C(t)]}{dt} \right| _{\text {ODE}}$ is computed using Eq. 19. To further facilitate NN convergence, we applied output scaling, a standard technique in NN training. Specifically, Min–Max scaling was applied to rescale the observed outputs to the 0–1 interval. The observed mean and standard deviation of the plasma concentration were transformed as follows:

33 $$\begin{aligned} X_{scaled} = \frac{X_{uncsaled} -X_{min}}{X_{max} - X_{min}} \end{aligned}$$

where $X_{min}$ and $X_{max}$ correspond to the minimum and maximum values observed for the mean and standard deviation of the plasma concentration. Since no data were available for the lymph, leaky and tight tissue concentrations, their outputs were scaled using the minimum and maximum values of the mean plasma concentration. To obtain the true parameter values and use them in the right-hand side of the ODE system, the NN outputs were subsequently mapped back to the original scale using the inverse transformation:

34 $$\begin{aligned} X_{unscaled} = X_{scaled}\cdot (X_{max} - X_{min}) + X_{min} \end{aligned}$$

Finally, because AD produces derivatives of the scaled variables while the ODE system is expressed in unscaled variables, these derivatives were mapped back to the original scale before computing the ODE loss term, using the following transformation:

35 $$\begin{aligned} \left. \frac{d \mathbb {E}[C(t)]_{\text {unscaled}}}{dt}\right| _{\text {AD}}= \left. \frac{d\mathbb {E}[C(t)]_{\text {scaled}}}{dt} \right| _{\text {ODE}} \\\cdot \left( \max \mathbb {E}[C(t)] - \min \mathbb {E}[C(t)] \right) \end{aligned}$$

36 $$\begin{array}{c} \left. \frac{d\textrm{SD}[C(t)]_{\text {unscaled}}}{dt} \right| _{\text {AD}}= \left. \frac{d\textrm{SD}[C(t)]_{\text {scaled}}}{dt} \right| _{\text {ODE}} \\ \cdot \left( \max \textrm{SD}[C(t)] - \min \textrm{SD}[C(t)] \right) ^{2} \end{array}$$

The unscaled standard deviation was internally converted to variance, and Algorithm 1 was followed. Similar to case study 1, at each iteration, the predictions of the NN along with the distributional parameters of $CL_p$ were used to construct the joint probability distribution. At each time point $t_j$, samples were drawn from the joint distribution over the plasma concentration and the $CL_p$ parameter, as described in Eq. 37.

37 $$\begin{aligned} {[}\log C^{(i)}_{plasma}(t_j), \log CL_{p_{j}}^{(i)}{]}&\sim \textrm{MVN}(\boldsymbol{\mu }\boldsymbol{'}_{\!\!\boldsymbol{j}}, \Omega _j') \end{aligned}$$

where $\boldsymbol{\mu _j'}$ and $\Omega _j'$ were constructed using log transformations similar to the ones described in the first case study. The reflection coefficients were parameterised to respect their physical constraints. Specifically, $\sigma _1$ was mapped to the interval (0, 1) using an inverse-logit transformation of an unconstrained auxiliary variable $\sigma _{1,raw}$, while $\sigma _2$ was defined as the product of $\sigma _1$ and the inverse-logit transformation of unconstrained auxiliary variable $\sigma _{2,raw}$. This ensured that both coefficients remained below 1 and that $\sigma _1 \ge \sigma _2$ at all times. Finally, a log-normal error model was applied to account for measurement error and derive the noise-free concentrations (Eq. 14).

The NN architecture was kept simple, consisting of two hidden layers with 30 neurons each, using the *tanh*() activation function. After a tuning procedure, the number of collocation points was set to 100, which ensured a dense grid over the time span of the simulation while keeping computational cost low. The number of virtual subjects was also set to 100. NN weights were initialised using the Glorot normal initialiser, and no loss regularisation was applied. The model was trained with the ADAM optimizer for 70000 iterations, with an initial learning rate of 5e-04 and an exponential decay schedule that reduced the learning rate by 1% every 1000 iterations. All loss terms contributed equally to the total loss by assigning a unit weight to each term.

#### Benchmarking with Markov Chain Monte Carlo (MCMC)

The strengths and weaknesses of a methodology can be assessed through comparison with alternative approaches. Towards this direction, case study 2 was also modelled using a Markov-chain Monte Carlo analogue introduced in [19]. Stan [24], which provides an efficient implementation of the NUTS algorithm [25], was used for modelling. The summarised concentration data were reanalysed under this framework. The same number of virtual subjects as in the D-PINN model was considered ($N_{samples}=100$), with each subject receiving a pre-assigned body weight sampled from a uniform distribution that was kept fixed for the entire simulation. At every iteration, individual clearance parameters and the two reflection coefficients were sampled and applied to solve the ODE system for each subject. At the study sampling times, measurement error was added, the simulated concentrations were aggregated using the mean and standard deviation and were finally compared against the reported summary statistics.

A three-tiered hierarchical model was employed to characterise the data. The first tier defined a likelihood on the summary statistics of the plasma concentrations, assuming log-normal observational errors for both the mean and the standard deviation. The second tier described the individual-level parameters, and the third tier covered the population level, assigning prior distributions to the population parameters. For the reflection coefficients, sampling was performed at the population level, whereas for clearance, a population mean and interindividual variability were used to sample individual clearance values.The statistical formulation is presented in Eqs. 38–46.

*First stage*

38 $$\begin{aligned} \log \!\big (\mathbb {E}[C(t_i)]\big )&\sim N\!\left( \log \!\big (\widehat{\mathbb {E}[C(t_i)]}\big ), \; \sigma _{\text {mean}}^2 \right) \end{aligned}$$

39 $$\begin{aligned} \log \!\big (\textrm{SD}[C(t_i)]\big )&\sim N\!\left( \log \!\big (\widehat{\textrm{SD}[C(t_i)]}\big ), \; \sigma _{\text {sd}}^2 \right) \end{aligned}$$

*Second stage*

For each subject *j*, clearance follows a lognormal distribution:

40 $$\begin{aligned} \begin{aligned} CL_j&= e^{\mu _{\textrm{CL}} + \tau _{\textrm{CL}}\, z_j}, \quad z_j \sim N(0,1), \end{aligned} \end{aligned}$$

The PBPK model produces individual predictions, $\hat{C}_k(t_i)$, and then measurement error is added:

41 $$\begin{aligned} C_k(t_i)&= \hat{C}_k(t_i)\cdot e^{\epsilon _{kj}}, \quad \epsilon _{kj} \sim N(0,\sigma ^2) \end{aligned}$$

The predicted mean across subjects is computed as:

42 $$\begin{aligned} \widehat{\mathbb {E}[C(t_i)]}&= \frac{1}{N_{\text {subj}}} \sum _{k=1}^{N_{\text {subj}}} C_k(t_i). \end{aligned}$$

The predicted standard deviation across subjects is:

43 $$\begin{aligned} \widehat{\textrm{SD}[C(t_i)]}&= \sqrt{ \frac{1}{N_{\text {subj}} - 1} \sum _{k=1}^{N_{\text {subj}}} \left( C_k(t_i) - \widehat{\mathbb {E}[C(t_i)]} \right) ^{2} }. \end{aligned}$$

The reflection coefficients were transformed similar to the D-PINN model as:

44 $$\begin{aligned} \sigma _1&= \textrm{logit}^{-1}(\sigma _{1,\text {raw}}), \end{aligned}$$

45 $$\begin{aligned} \sigma _2&= \sigma _1 \cdot \textrm{logit}^{-1}(\sigma _{2,\text {raw}}). \end{aligned}$$

*Third stage*

46 $$\begin{aligned} \begin{aligned} \mu _{\textrm{CL}}&\sim N\!\left( \mu _{\textrm{CL}}^{*},\; \sigma _{\mu _{\textrm{CL}}}^{*}\right) \\ \tau _{\textrm{CL}}&\sim N_{half}\!\left( \tau _{\textrm{CL}}^{*},\; \sigma _{\tau _{\textrm{CL}}}^{*}\right) \\ \sigma _{\text {mean}}&\sim N_{half}(0,1) \\ \sigma _{\text {sd}}&\sim N_{half}(0,1) \\ \sigma _{1,\text {raw}}&\sim N(0,1) \\ \sigma _{2,\text {raw}}&\sim N(0,1) \end{aligned} \end{aligned}$$

where $N_{half}$ is the half normal distribution. The transformed hyperparameters $\mu _{\textrm{CL}}^{*}, \sigma _{\mu _{\textrm{CL}}}^{*}$ and $\tau _{\textrm{CL}}^{*}, \sigma _{\tau _{\textrm{CL}}}^{*}$ are computed in the transformed data block to map priors specified on the normal scale to the log scale.

### Software and code availability

All models were run on a computer with an Intel Core i7-8700 (3.2 GHz), 16 GB RAM and windows 10 OS. All computations relevant to D-PINNs were facilitated using the python package DeepXDE (v.1.9.3) [26], and in particular the Tensorflow (v.2.1.3) interface [27]. Stan was implemented through the RStan (v.2.17.3) interface [28]. The code used in this work is available at: https://github.com/ntua-unit-of-control-and-informatics/d-pinns-pharmacokinetics

## Results and discussion

### Case study 1

The first case study aimed to demonstrate the ability of the D-PINNs methodology to recover the population-level distribution parameters of the ODE parameters using only aggregated plasma concentration data. The method was applied to a simple, one-compartment pharmacokinetic model with simulated data, chosen for its clarity and interpretability as an illustrative example. To evaluate and better understand model performance and limitations, a series of plots were generated. Unless explicitly stated otherwise, predictions were generated using $N_{\text {samples}} = 100$, $N_{cp} = 100$, equally spaced within the time domain, and unit loss weights, while the corresponding simulated data were generated using zero correlation between $V_d$ and $k_e$, denoted by $\rho _{V_d, k_e}$. All models converged within 30,000 iterations, and all reported results are based on training up to this point.

#### Model performance assessment and parameter inference

**Fig. 6:**
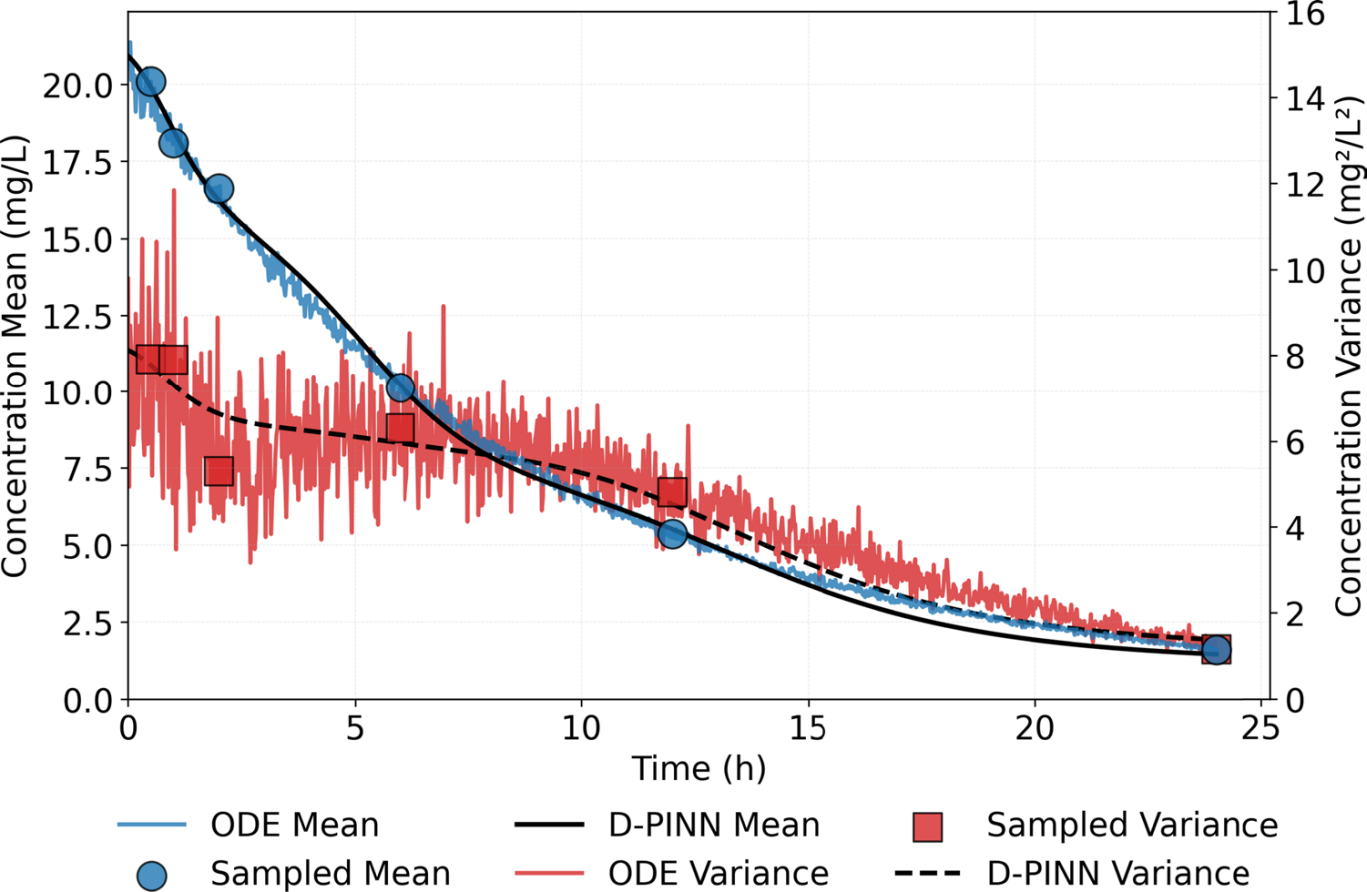
Comparison between the predicted mean and variance of the plasma concentration against the corresponding observed values. The full simulated time series of the simulated mean and variance are also depicted on the graph

Figure 6 compares the NN predictions to sampled observations. To provide additional insights to model dynamics, the full simulated time series for the mean and variance are also included in the figure. These time series reveal that empirical variance estimates are more sensitive to the sampled measurement noise, while empirical mean estimates are less affected. The NN prediction curves closely follow the sampled concentration means and variances, with the model avoiding overfitting, i.e., it does not mirror fluctuations in the sampled data arising from sampling noise, particularly in early time points where variance tends to be more unstable. The results highlight the value of physics-based constraints. In regions lacking observation points, for example in $6-12 \, \text {h}$ and $12-24 \,\text {h}$ intervals, model predictions remain aligned with the underlying ODE solution shape, suggesting that the ODE loss terms guide the network toward physiologically plausible behavior even in the absence of data.

**Fig. 7:**
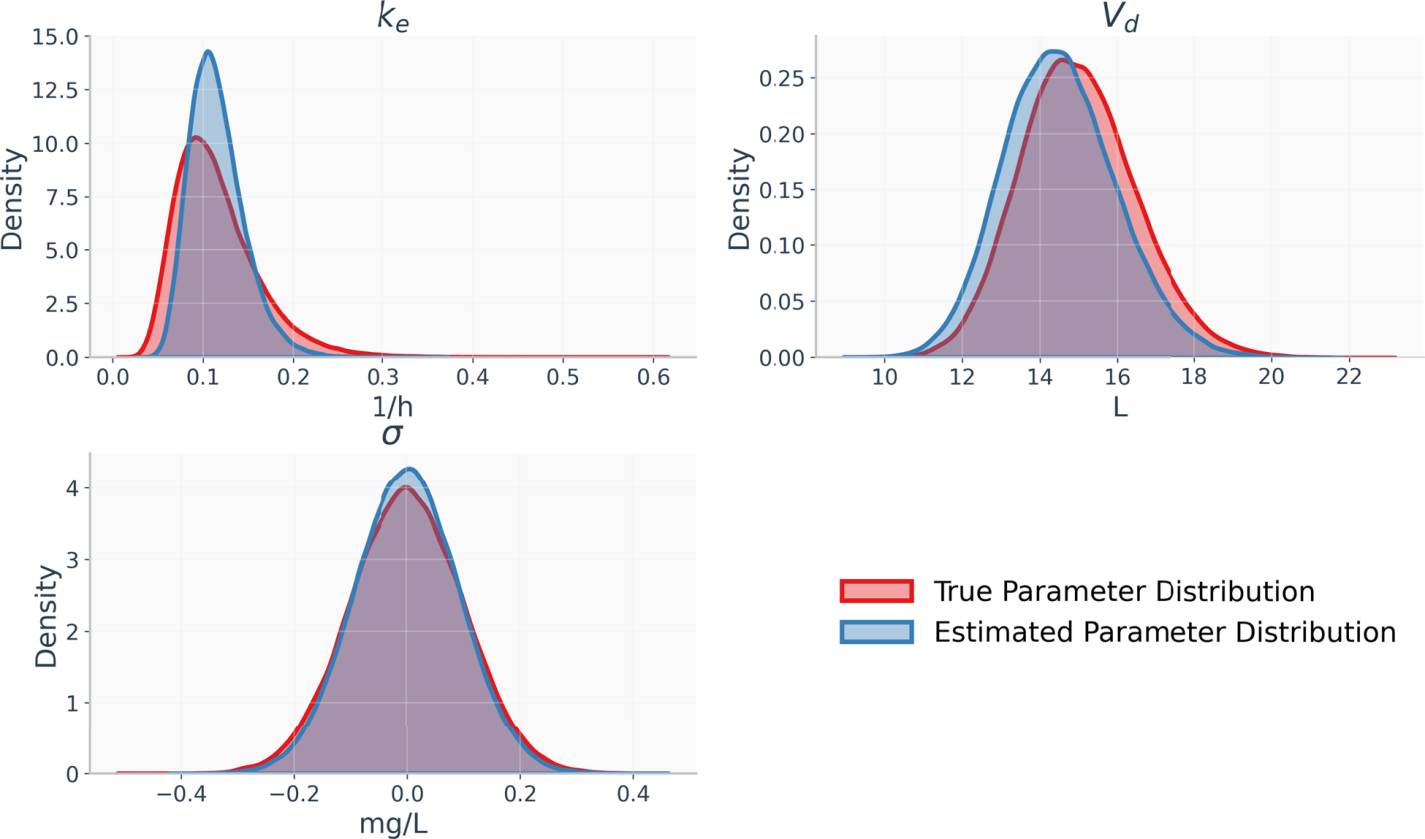
Comparison of population parameter distribution and predicted parameter distribution using the parameter estimates from the D-PINN model. Both distributions were generated using 100,000 samples

The true population-level parameter distribution along with the distribution derived from the D-PINN are illustrated in Fig. 7. Each distribution is represented by the density of 100,000 samples. The true distribution is based on the predefined parameter values used during data simulation, while the predicted distribution corresponds to the parameters estimated by the D-PINNs algorithm. The density plot of $k_e$ reveals that the D-PINN slightly underestimates the variance of this parameter. This deviation from true values should be expected for parameters that exhibit high population variability, as is the case for $k_e$.

Detailed parameter estimates, along with the ground-truth population values are provided in Table 1. The model recovers the parameters associated with $V_d$, the mean of $k_e$, as well as the measurement noise parameter $\sigma$ corresponding to the log-normal error model with high accuracy. The recovery of the variance of $k_e$ was less accurate. This deviation from the true value can be attributed to the high interindividual variability of $k_e$. Interestingly, the model estimates the population mean and variance of $V_d$ with high precision. In this setup, $V_d$ is most informative at $t_0 = 0$, where the concentration is defined by the initial condition $C(t_0) = \frac{\text {Dose}}{V_d}$. The initial condition loss is computed as the MSE between model predictions at $t_0$ and sampled values of $\frac{\text {Dose}}{V_d}$. The accurate recovery of both the mean and variance of $V_d$ thus demonstrates the effectiveness of the constraints imposed by the initial condition loss.

**Table 1:** True and D-PINN estimates of population-level pharmacokinetic parameters. The symbols $\mu _{k_e}$, $\sigma _{k_e}$, $\mu _{V_d}$, and $\sigma _{V_d}$ denote the mean and standard deviation of $k_e$ and $V_d$, respectively, while $\sigma$ represents the residual standard deviation on the log scale

	$\mu _{k_e}$	$\sigma _{k_e}$	$\mu _{V_d}$	$\sigma _{V_d}$	$\sigma$
True population values	0.116	0.046	15.000	1.500	0.100
Estimated population values	0.115	0.030	14.530	1.462	0.093

#### D-PINN hyperparameter tunning

As shown in Eq. 7, multiple loss weights are incorporated into the loss function, and these weights require careful tuning. All results presented so far assume unit loss weights. Table 2 summarises the impact of assigning different weights to loss terms on the total loss and on MAPE of the estimates. Increasing the loss weights led to a slight reduction in both the total loss and MAPE, with the effect being more pronounced when the weights associated with the initial condition terms were increased. The lowest MAPE was observed when unit weight losses were assumed. Testing the use of separate weights for the expected value and variance terms (not shown here) did not reveal any systematic trend. Nonetheless, loss weight selection is a known challenge in the training of PINNs, where model performance can be sensitive to the choice of loss weights. To address this issue, self-adaptive loss weight adjustment techniques have been proposed [29]. It is important to note that in this study, the true parameter values were known, allowing MAPE to serve as a direct evaluation metric. In real-world applications, where true parameter values are not known, the impact of loss weight selection would instead be assessed based on the model’s ability to fit the observed clinical data and produce physiologically plausible parameter estimates.

**Table 2:** Influence of loss weight selection on the total D-PINN loss ($\mathcal {L}_{\text {total}}$) and the mean absolute percentage error (MAPE) of the D-PINN estimates relative to the true population values

$\lambda _{\text {ODE}}^{\mathbb {E}}$	$\lambda _{\text {ODE}}^{\textrm{Var}}$	$\lambda _{\text {IC}}^{\mathbb {E}}$	$\lambda _{\text {IC}}^{\textrm{Var}}$	$\lambda _{\text {D}}^{\mathbb {E}}$	$\lambda _{\text {D}}^{\textrm{Var}}$	$\mathcal {L}_{\text {total}}$	MAPE (%)
1	1	1	1	1	1	1.0	9.4
5	5	1	1	1	1	1.1	9.9
10	10	1	1	1	1	1.3	10.3
20	20	1	1	1	1	1.5	9.5
50	50	1	1	1	1	5.4	11.0
1	1	5	5	1	1	5.0	14.8
1	1	10	10	1	1	7.3	16.2
1	1	20	20	1	1	14.9	12.7
1	1	50	50	1	1	36.5	17.1
1	1	1	1	5	5	1.7	10.8
1	1	1	1	10	10	2.6	12.2
1	1	1	1	20	20	4.2	10.0
1	1	1	1	50	50	9.2	17.0

**Fig. 8:**
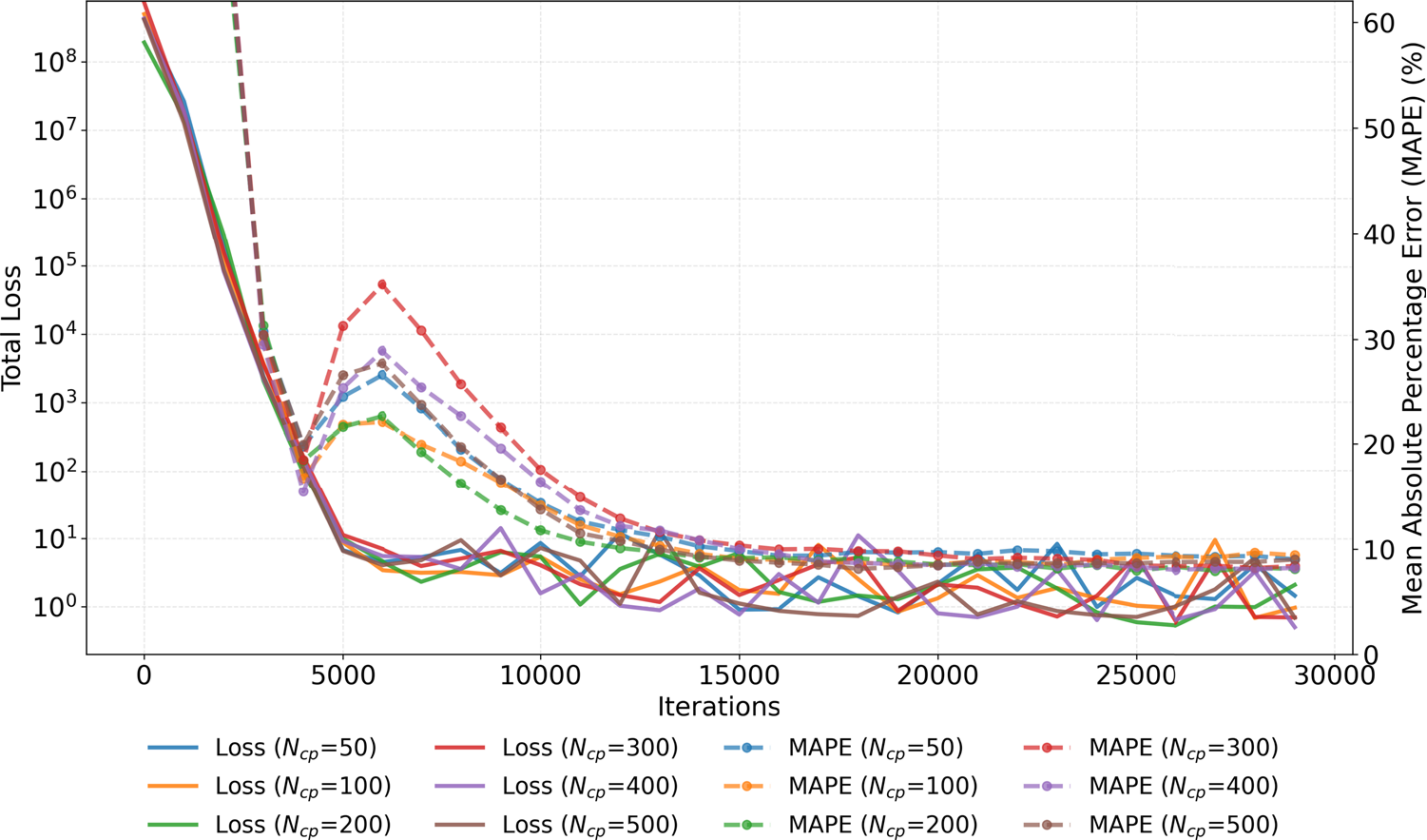
Evolution of the mean absolute percentage error (MAPE) of the D-PINN model with respect to the true parameter values (dashed line) along with evolution of the total loss (solid line), for different number of collocation points, $N_{cp}$, used for computing the ODE loss terms. Simulated data were generated using parameter correlation $\rho _{k_e, \, V_d} = 0$, and the model used $N_{\text {samples}} = 100$ and unit loss weights

An important parameter in model calibration is the number of collocation points, $N_{cp}$. Figure 8 illustrates the effect of varying $N_{cp}$ on the total D-PINN loss and on MAPE. Increasing $N_{cp}$ did not lead to a noticable reduction in MAPE. Across all tested values, the corresponding total losses were also comparable. Internal tests indicated that the observed variability in total loss is primarily due to sampling variability, which particularly affects the initial condition variance loss term $\mathcal {L}_{\text {IC}}^{\textrm{Var}}$. Finally, the execution time increased with increasing $N_{cp}$, with $N_{cp} = 50, 100, 200, 300, 400,$ and 500 collocation points requiring 160, 170, 231, 280, 300, and 310 seconds, respectively.

**Fig. 9:**
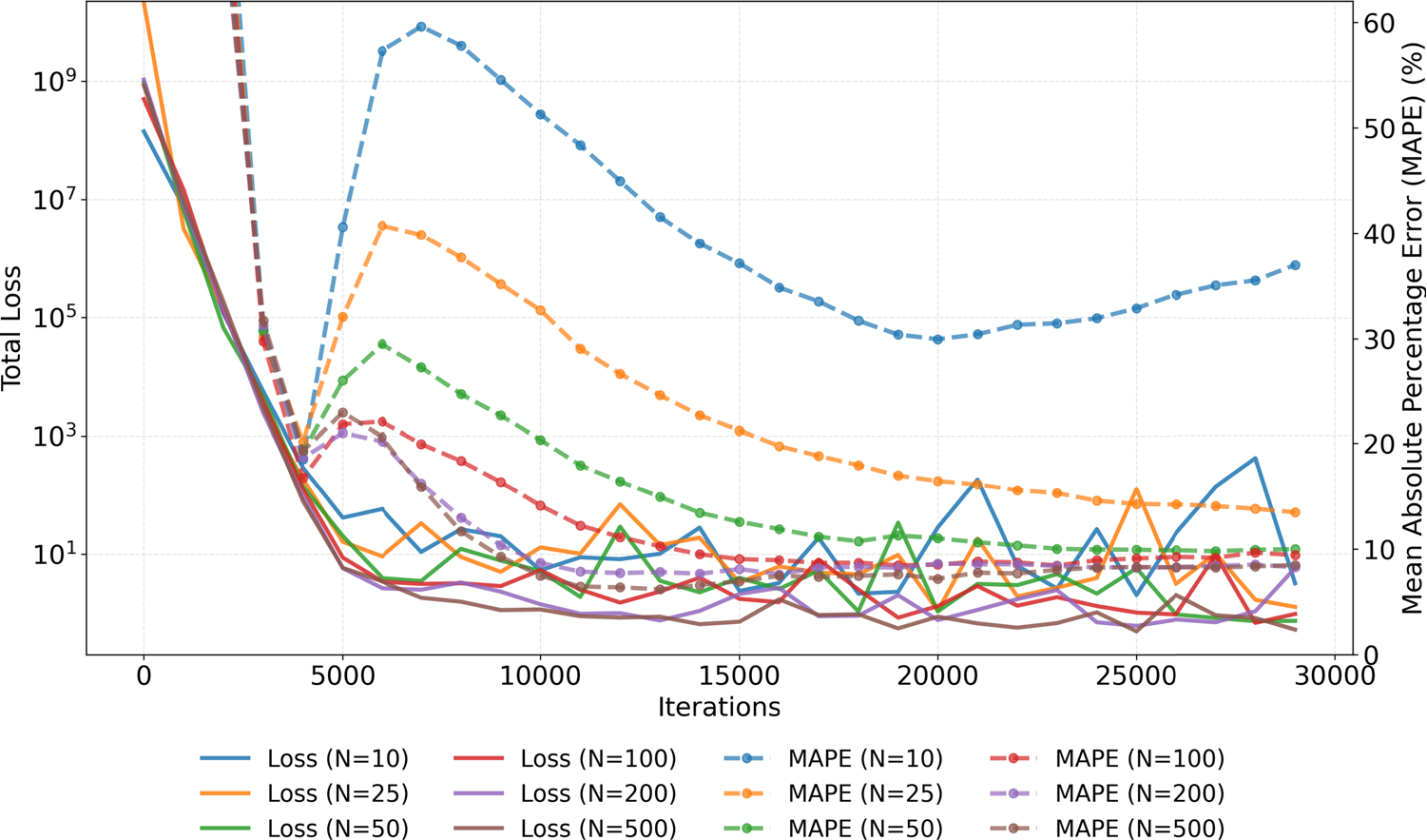
Evolution of the mean absolute percentage error (MAPE) of the D-PINN model with respect to the true parameter values (dashed line) along with evolution of the total loss (solid line), for different number of samples, $N_{\text {samples}}$, used for computing the ODE loss terms. Simulated data were generated using parameter correlation $\rho _{k_e, \, V_d} = 0$, and the model used $N_{cp} = 300$ and unit loss weights

Figure 9 demonstrates the effect of $N_{\text {samples}}$ on the total loss and on MAPE. Convergence was achieved within 30, 000 iterations across all tested values of $N_{\text {samples}}$, except for $N_{\text {samples}} = 10$. Increasing $N_{\text {samples}}$ consistently resulted in lower MAPE values, reduced total loss, and decreased variability in the total loss. The execution times for $N_{\text {samples}} = 10$, 25, 50, 100, 200, and 500 samples was 107, 110, 115, 141, 183, and 329 seconds, respectively. As also observed in Fig. 8, the minimisation of both the total loss and MAPE tends to occur in parallel, with lower total loss typically accompanied by lower MAPE. However, it is important to note that the accuracy of parameter estimation should not be confused with the total D-PINN loss. The latter reflects the sum of the ODE, data, and initial condition loss terms, and its minimisation does not necessarily yield the most accurate parameter estimates. Interestingly, both Figs. 8 and 9 exhibit an elbow-like behaviour, where a reasonable solution is reached before full convergence, followed by a temporary increase in MAPE before it decreases again.

#### Sensitivity to simulation parameters

**Fig. 10:**
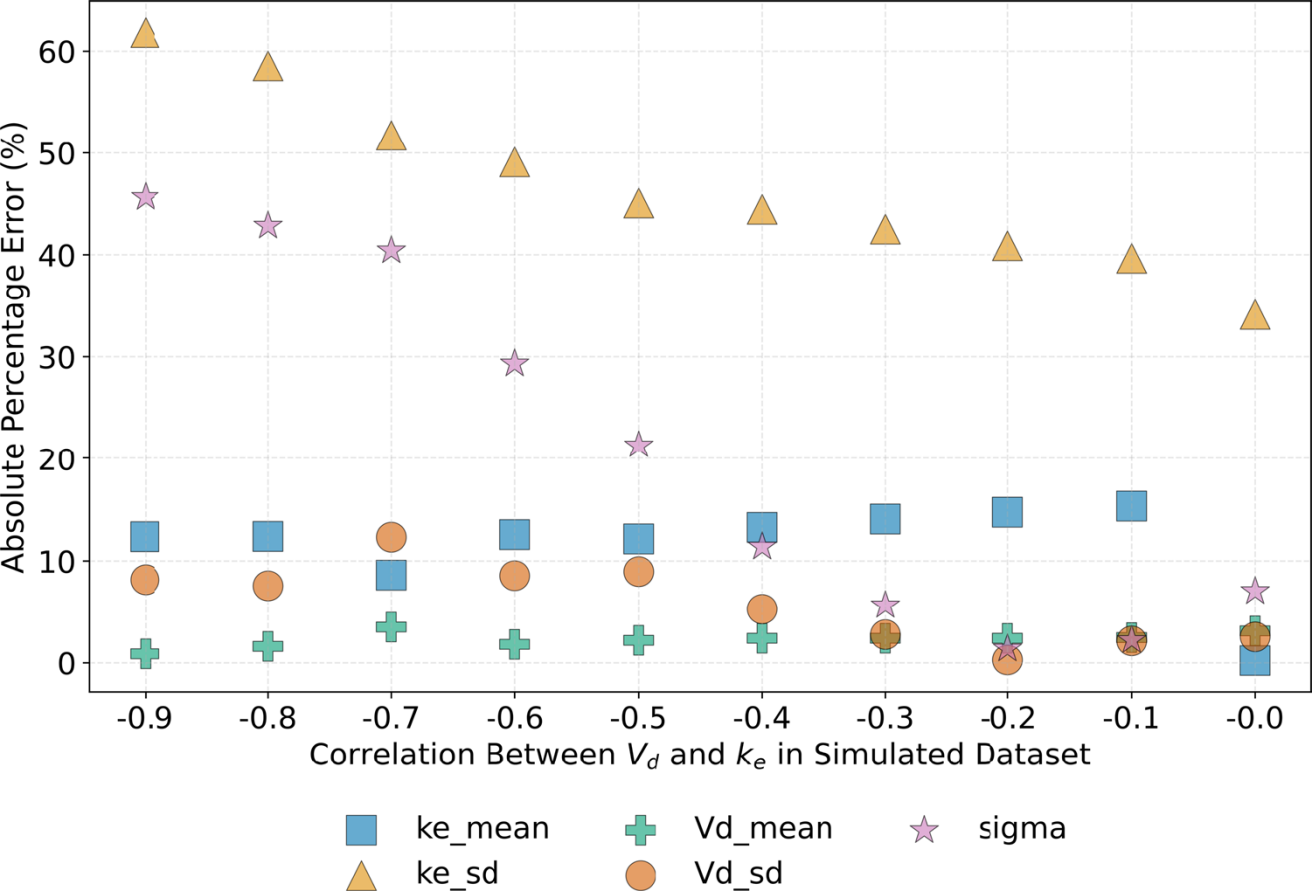
Impact of varying parameter correlation values, $\rho _{k_e, V_d}$, on the accuracy of D-PINN parameter estimation. The model was configured with $N_{cp} = 100$, $N_{\text {samples}} = 100$, and unit loss weights

To assess the impact of parameter correlation on the ability of D-PINNs to recover the underlying parameter distributions, ten different simulation scenarios were constructed with varying correlation values, and the model was repeatedly fitted to the resulting data. To ensure comparability across correlation scenarios, the same random residual errors were pre-generated and consistently applied to each virtual patient and timepoint in all simulations. The outcomes are summarised in Fig. 10. As the absolute value of the correlation increases, the accuracy of parameter recovery decreases. Nevertheless, the results remain within an acceptable range. The parameters most strongly affected by correlation between $V_d$ and $k_e$ are the resisual standard error, followed by the standard deviation of $k_e$.

Note that neither Table 1 nor Fig. 10 include the correlations between the ODE parameters, or between the parameters and the state variable. In the absence of individual concentration-time profiles, correlation parameters can’t be reliably inferred from summary statistics alone. Across all simulations, the optimisation consistently yielded similar values for the correlation parameters, regardless of the true input correlation. Specifically, $\rho _{V_d, k_e}$ was repeatedly estimated near the lower bound of the correlation spectrum, while the correlations between the parameters and plasma concentration remained close to zero. These values were not included in the plots, as estimating correlation structure lies outside the intended scope of D-PINNs and was not considered essential for evaluating the methodology. However, when we tested a version of the model with the correlations manually fixed to zero, we observed a slight deterioration in both the model loss and the accuracy of parameter estimates across datasets with varying true correlation levels. Although the correlation between parameters or between parameters and states cannot be estimated using only the mean and variance of the concentration, the D-PINN framework also allows this quantity to be fixed rather than calibrated. This enables sensitivity analysis by evaluating model performance under different assumed correlation values, either through systematic variation or by incorporating prior estimates from the literature.

**Fig. 11:**
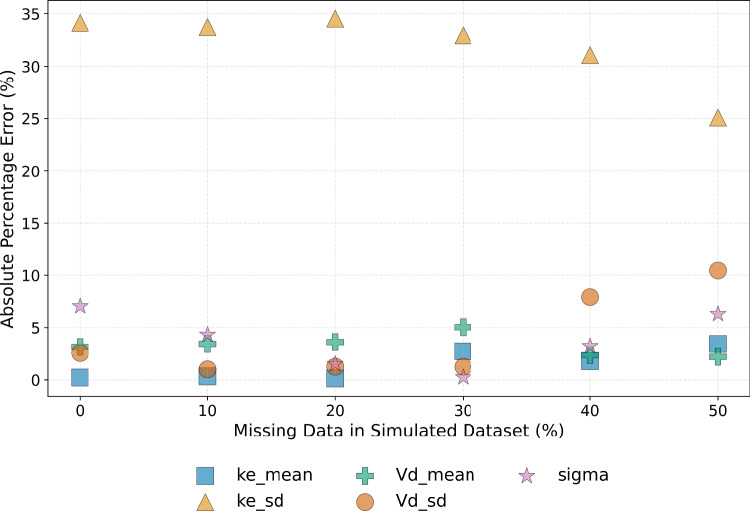
Impact of the percentage of missing observations on D-PINN parameter estimation accuracy. Simulated data were generated using $\rho _{k_e, V_d} = 0.0$, and the model was configured with $N_{cp} = 100$, $N_{\text {samples}} = 100$, and unit loss weights

Finally, Fig. 11 illustrates the impact of missing observations on parameter estimation accuracy. To generate this plot, a predefined percentage of data points was randomly removed across patients and time points to simulate missing observations due to incomplete sampling. Even with $50\%$ of the observations missing, the model maintains a high level of estimation accuracy. This finding suggests that population-level pharmacokinetic parameter distributions could be reliably inferred even when fewer samples per patient are available.

### Case study 2

The second case study focused on demonstrating the application of D-PINNs for parameter estimation in an mPBPK model using aggregated mAb plasma data. Apart from D-PINNs, an MCMC analogue was also used to model the available data. Estimation of both the interindividual variability in clearance and the measurement error proved less stable in this example for both frameworks, so the standard deviation of the noise was fixed at 0.1. After fixing the dispersion of the measurement error, running Stan diagnostics did not reveal any pathological behaviour, and R-hat values were approximately 1 for all parameters. The D-PINN model converged within 70,000 iterations, with an execution time of approximately 400 seconds, which is comparable to that of the one-dimensional ODE system in case study 1. The MCMC implementation used four parallel chains with 1000 iterations in total, of which 400 were discarded as warm-up, and the execution time was roughly 5000 seconds. This comparison is intended only to provide an indication of computational scale. For consistency, the same number of virtual subjects was used as in the D-PINN implementation, even though MCMC solves the ODE system directly at each iteration. In practice, comparable results could be achieved with fewer virtual subjects, which would substantially reduce the MCMC computational burden. Moreover, the MCMC execution time could be further reduced by decreasing the number of iterations.

The estimated parameters from the D-PINNs methodology are reported in Table 3 along with the values estimated by MCMC. The values estimated using D-PINNs closely matched those obtained with MCMC, and in both cases the values were consistent with the ranges reported for other mAbs in [23]. A slightly higher standard deviation of $CL_p$ was observed in the D-PINN results, along with small deviations in the reflection coefficients.

**Table 3:** Fitted mPBPK model parameters using D-PINNs and MCMC

Parameters	D-PINNs	MCMC
$\sigma _1$	0.8378	0.8835
$\sigma _2$	0.7529	0.6657
$\mu _{CL_p}$	0.0115	0.0119
$\sigma _{CL_p}$	0.0041	0.0032

**Fig. 12:**
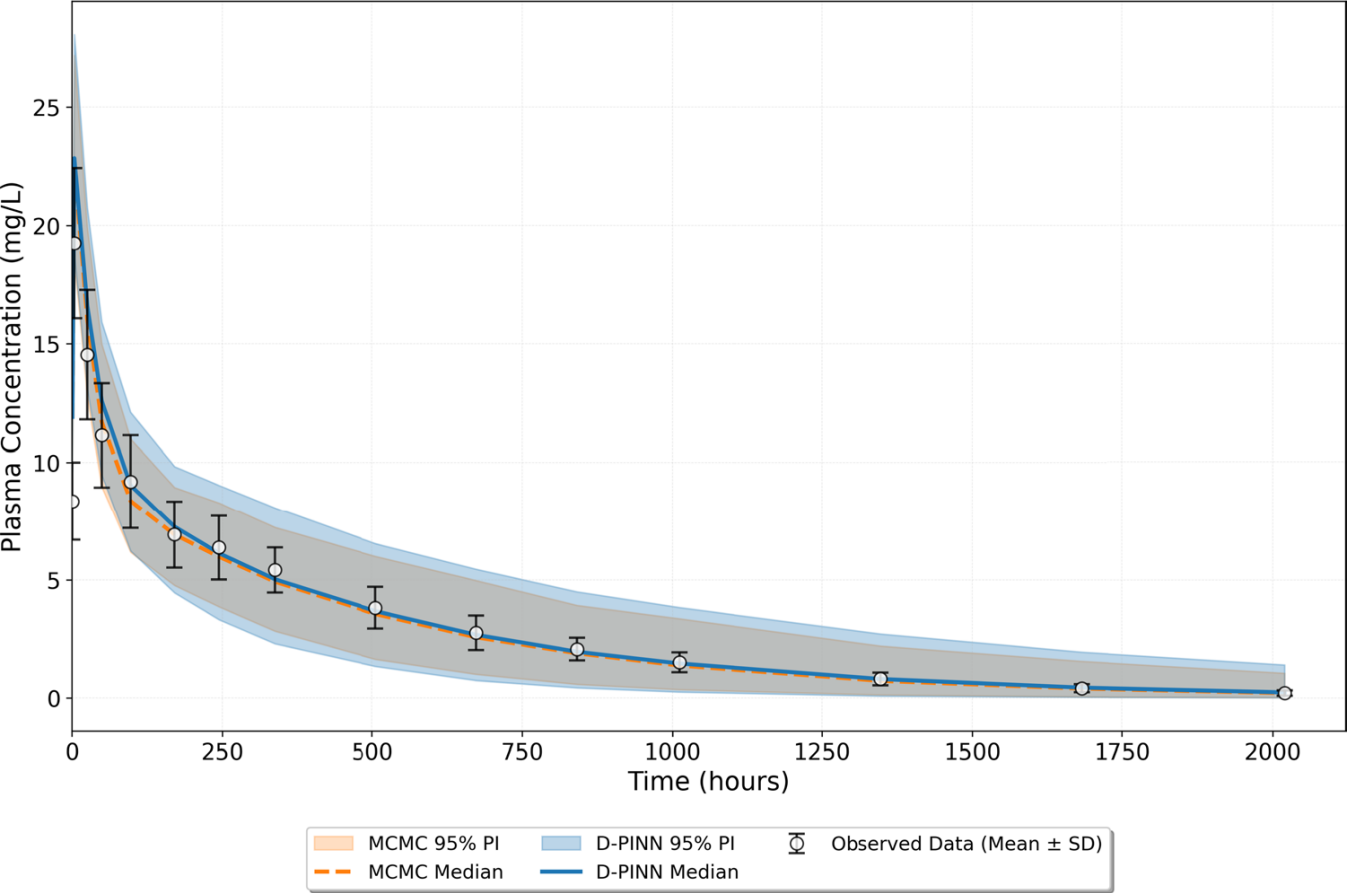
Comparison of the D-PINN and MCMC model predictions against TAB008 plasma concentration summary statistics. The observed mean data are plotted as open circles with error bars representing the standard deviation (SD). The solid blue and dashed orange lines represent the median predictions, while the blue and orange bands represent the 95% Prediction Intervals of the D-PINN and MCMC models, respectively

The estimated parameters obtained using both approaches were used in a Monte Carlo simulation to evaluate whether the resulting models accurately capture the observed central tendency and variability observed in the TAB008 data. Specifically, $CL_p$ and body weight were sampled 1000 times from their respective distributions. The ODE system was then solved for each sampled parameter set and measurement error was introduced using a proportional error with a standard deviation of 0.1. The results of this Monte Carlo simulation are presented as median predictions with 95% prediction intervals, compared against the mean and standard deviation of the experimental data in Fig. 12. The plot demonstrates strong agreement between the D-PINN and MCMC predictions. The median profiles from both methods closely follow the experimental observations across all time points. Both approaches captured the central tendency well, although the predicted variability was slightly overestimated at later time points, which is likely due to limited information content in the summarised data. The D-PINN framework exhibits slightly wider prediction intervals, which is consistent with the marginally higher estimated standard deviation for $CL_p$ relative to the MCMC results.

## Conclusions

In this work, we introduced D-PINNs, a novel extension of the PINN framework designed to estimate population-level pharmacokinetic parameters from aggregated concentration data. Unlike traditional approaches that rely on individual concentration-time profiles, D-PINNs use reported mean and variance values to reconstruct the underlying parameter distributions. This makes them particularly suitable for meta-analysis of published studies where only summary statistics are available. Through a simulated one-compartment pharmacokinetic model, we demonstrated that D-PINNs can accurately recover the distributions of key model parameters, including both interindividual variability and residual measurement noise. The framework showed strong agreement between predicted and true parameter values when the underlying model structure and distributional assumptions were correctly specified. The application of the methodology to reported mAb aggregated concentration data demonstrated the capability of D-PINNs to address real world problems, yielding similar performance with an MCMC-based analogue. Overall, this work presents a proof-of-concept showing that D-PINNs provide a flexible and effective approach for parameter inference in the absence of individual-level data. Future work should focus on testing the methodology to more complex systems and on extending the methodology to incorporate sample size information to better address the uncertainty and variability present in real-world applications.

## Data Availability

No datasets were generated or analysed during the current study.

## Appendix A Derivation of the concentration mean and concentration variance derivatives

We define the state variable $\boldsymbol{C}(t) = h(t, \boldsymbol{\eta })$, where $\boldsymbol{\eta }$ is a random vector of ODE parameters drawn from a time-invariant probability density function $g(\boldsymbol{\eta }; \boldsymbol{\theta }_{\eta })$, parameterized by the distributional parameters $\boldsymbol{\theta }_{\eta }$. Since the distribution is fixed over time, we have $\frac{d}{dt} g(\boldsymbol{\eta }; \boldsymbol{\theta }_{\eta }) = 0$.

The expected value of the concentration vector at time *t* is given by:

47 $$\begin{aligned} \mathbb {E}[\boldsymbol{C}(t)] = \int _{\boldsymbol{\eta }} h(t, \boldsymbol{\eta }) \, g(\boldsymbol{\eta }; \boldsymbol{\theta }_{\eta }) \, d\boldsymbol{\eta } \end{aligned}$$

The following derivation assumes that the regularity conditions required to apply Leibniz’s rule for differentiation under the integral sign are satisfied. Specifically, we assume that the integrand is sufficiently smooth and that the necessary integrability conditions hold. These assumptions allow the interchange of differentiation and integration. Thus, differentiating Eq. 47 with respect to time and applying the Leibniz rule yields:

48 $$\begin{aligned} \frac{d \mathbb {E}[\boldsymbol{C}(t)]}{dt}&= \frac{d}{dt} \int _{\boldsymbol{\eta }} h(t, \boldsymbol{\eta }) \, g(\boldsymbol{\eta }; \boldsymbol{\theta }_{\eta }) \, d\boldsymbol{\eta } \nonumber \\&= \int _{\boldsymbol{\eta }} \frac{d h(t, \boldsymbol{\eta })}{dt} \, g(\boldsymbol{\eta }; \boldsymbol{\theta }_{\eta }) \, d\boldsymbol{\eta } \nonumber \\&= \mathbb {E}\left[ \frac{d \boldsymbol{C}(t)}{dt} \right] \end{aligned}$$

Therefore, the derivative of the mean can be approximated using the empirical mean formula:

49 $$\begin{aligned} \frac{d\,\widehat{\mathbb {E}[C(t)]}}{dt} = \frac{1}{N_{\text {samples}}} \sum _{i=1}^{N_{\text {samples}}} \frac{d\,C^{(i)}(t)}{dt} \end{aligned}$$

where $\frac{d\,C^{(i)}(t)}{dt}$ is the simulated derivative at time *t*, corresponding to the *i*-th sample of $\boldsymbol{\eta }$ and $\boldsymbol{C}(t)$.

Similarly, the empirical variance of the concentration at time *t* is defined as:

50 $$\begin{aligned} \widehat{\textrm{Var}[C(t)]} = \frac{1}{N_{\text {samples}} - 1} \sum _{i=1}^{N_{\text {samples}}} \left( C^{(i)}(t) - \widehat{\mathbb {E}[C(t)]} \right) ^2 \end{aligned}$$

Its derivative with respect to time is given by:

51 $$\begin{aligned} \frac{d\,\widehat{\textrm{Var}[C(t)]}}{dt}&= \frac{2}{N_{\text {samples}} - 1} \sum _{i=1}^{N_{\text {samples}}} \left( C^{(i)}(t) - \widehat{\mathbb {E}[C(t)]} \right) \cdot \nonumber \\&\hspace{4em} \left( \frac{d\,C^{(i)}(t)}{dt} - \frac{d\,\widehat{\mathbb {E}[C(t)]}}{dt} \right) \end{aligned}$$

## Appendix B Derivation of the relationship between the variance of observed and true concentrations

We consider the log-normal observation model for time point *j*:

52 $$\begin{aligned} C(t) = \boldsymbol{C}^{\text {true}}(t) \cdot e^{\varepsilon }, \quad \varepsilon \sim \mathcal {N}(0, \sigma ^2) \end{aligned}$$

Concentrations $\boldsymbol{C}^{\text {true}}(t)$ and *C*(*t*) are random variables. The aim here is to derive the relationship between the time derivatives of the expected value and variance of the observed and denoised (true) concentrations. Since the measurement error is assumed to follow a log-normal distribution, its mean and variance are known from standard log-normal formulas:

53 $$\begin{aligned} \mathbb {E}[e^{\varepsilon }]&= e^{\sigma ^2/2}, \end{aligned}$$

54 $$\begin{aligned} \textrm{Var}[e^{\varepsilon }]&= \left( e^{\sigma ^2} - 1 \right) \cdot e^{\sigma ^2} \end{aligned}$$

The above expressions are used in the derivation of the time derivatives of the mean and variance of the observed concentrations. Assuming independence between the measurement noise and the true concentration, it follows that:

55 $$\begin{aligned} \mathbb {E}[C(t)]&= \mathbb {E}[C^{\text {true}}(t)] \cdot \mathbb {E}[e^{\varepsilon }] \end{aligned}$$

56 $$\begin{aligned}&= \mathbb {E}[C^{\text {true}}(t)] \cdot e^{\sigma ^2/2} \end{aligned}$$

Differentiating with respect to time yields:

57 $$\begin{aligned} \frac{d \mathbb {E}[C(t)]}{dt}&= e^{\sigma ^2/2} \cdot \frac{d\mathbb {E}[C^{\text {true}}(t)]}{dt} \end{aligned}$$

This corresponds to the ODE-based derivative:

58 $$\begin{aligned} \left. \frac{d \mathbb {E}[C(t)]}{dt} \right| _{\text {ODE}}&= e^{\sigma ^2/2} \cdot \left. \frac{d\mathbb {E}[C^{\text {true}}(t)]}{dt} \right| _{\text {ODE}}. \end{aligned}$$

The identity for the variance of the product of two independent random variables is the following:

59 $$\begin{aligned} \textrm{Var}(XY) = \mathbb {E}[X]^2 \cdot \textrm{Var}(Y) + \mathbb {E}[Y]^2 \cdot \textrm{Var}(X) + \textrm{Var}(X) \cdot \textrm{Var}(Y), \end{aligned}$$

Based on the above, the variance of the observed concentration at time *t* becomes:

60 $$\begin{aligned} \textrm{Var}[C(t)]&= \mathbb {E}[C^{\text {true}}(t)]^2 \cdot \textrm{Var}[e^{\varepsilon }] + \mathbb {E}[e^{\varepsilon }]^2 \cdot \textrm{Var}[C^{\text {true}}(t)] \end{aligned}$$

61 $$\begin{aligned}&\quad + \textrm{Var}[C^{\text {true}}(t)] \cdot \textrm{Var}[e^{\varepsilon }] \nonumber \\&= e^{\sigma ^2} \cdot \left[ \mathbb {E}[C^{\text {true}}(t)]^2 \left( e^{\sigma ^2} - 1 \right) + \textrm{Var}[C^{\text {true}}(t)] \cdot e^{\sigma ^2} \right] \end{aligned}$$

Taking the derivative with respect to time yields:

62 $$\begin{aligned} \frac{d\textrm{Var}[C(t)]}{dt}&= e^{\sigma ^2} \cdot \big [\left( e^{\sigma ^2} - 1 \right) \cdot 2 \cdot \mathbb {E}[C^{\text {true}}(t)] \cdot \frac{d\mathbb {E}[C^{\text {true}}(t)]}{dt} \end{aligned}$$

63 $$\begin{aligned}&\quad + e^{\sigma ^2} \cdot \frac{d\textrm{Var}[C^{\text {true}}(t)]}{dt} \big ] \end{aligned}$$

This corresponds to the ODE-based derivative:

64 $$\begin{aligned} \left. \frac{d\textrm{Var}[C(t)]}{dt} \right| _{\text {ODE}}&= e^{\sigma ^2} \cdot \left[ \left( e^{\sigma ^2} - 1 \right) \cdot 2 \cdot \mathbb {E}[C^{\text {true}}(t)] \cdot \left. \frac{d\mathbb {E}[C^{\text {true}}(t)]}{dt} \right| _{\text {ODE}} \right. \nonumber \\&\quad \left. + e^{\sigma ^2} \cdot \left. \frac{d\textrm{Var}[C^{\text {true}}(t)]}{dt} \right| _{\text {ODE}} \right] \end{aligned}$$

## Appendix C Equations of the minimal physiologically-based pharmacokinetic model

Equations 65–72 are used to calculate $L_{1}$, $L_{2}$, $V_{tight}$, and $V_{leaky}$ [23].

65 $$\begin{aligned} L_1&= 0.33 \cdot L \end{aligned}$$

66 $$\begin{aligned} L_2&= 0.67 \cdot L \end{aligned}$$

67 $$\begin{aligned} V_{tight}&= 0.65 \cdot V_{ISF} \cdot K_p \end{aligned}$$

68 $$\begin{aligned} V_{leaky}&= 0.35 \cdot V_{ISF} \cdot K_p \end{aligned}$$

69 $$\begin{aligned} V_{blood}&= 0.073 \cdot BW \end{aligned}$$

70 $$\begin{aligned} V_{ISF}&= 0.15 \cdot BW \end{aligned}$$

71 $$\begin{aligned} V_{plasma}&= V_{blood} \cdot (1-Hct) \end{aligned}$$

72 $$\begin{aligned} V_{lymph}&= V_{blood} \end{aligned}$$

where $V_{ISF}$ is the total ISF volume, *Hct* is the hematocrit, considered equal to 0.43 for male volunteers [30] and $K_p$ represents the fraction of *ISF* that is available for mAb distribution, which is mainly defined by the antibody’s charge. Here, $K_p$ was fixed at 0.8 for native IgG1 antibodies, as reported in [23]. The total lymph flow was set to 3 L/day and $\sigma _L$ was set to 0.2 [23]. The fraction of the blood volume to body weight was set to 0.073 [31], while the fraction of ISF is equal to 0.15 [32].
